# Racial/Ethnic Disparities in Mortality Related to Access to Care for Major Cancers in the United States

**DOI:** 10.3390/cancers14143390

**Published:** 2022-07-12

**Authors:** Fei Wang, Xiang Shu, Tuya Pal, Jordan Berlin, Sang M. Nguyen, Wei Zheng, Christina E. Bailey, Xiao-Ou Shu

**Affiliations:** 1 Division of Epidemiology, Department of Medicine, Vanderbilt Epidemiology Center, Vanderbilt-Ingram Cancer Center, Vanderbilt University Medical Center, Nashville, TN 37203, USA or fei.wang@vumc.org (F.W.); sang.m.nguyen@vanderbilt.edu (S.M.N.); or wei.zheng@vumc.org (W.Z.); 2 Department of Breast Surgery, The Second Hospital, Cheeloo College of Medicine, Shandong University, Jinan 250033, China; 3 Department of Epidemiology & Biostatistics, Memorial Sloan Kettering Cancer Center, New York, NY 10065, USA; shux@mskcc.org; 4 Division of Genetic Medicine, Department of Medicine, Vanderbilt Genetics Institute, Vanderbilt-Ingram Cancer Center, Vanderbilt University Medical Center, Nashville, TN 37203, USA; tuya.pal@vumc.org; 5 Division of Hematology and Oncology, Department of Medicine, Vanderbilt-Ingram Cancer Center, Vanderbilt University Medical Center, Nashville, TN 37203, USA; jordan.berlin@vumc.org; 6 Division of Surgical Oncology and Endocrine Surgery, Department of Surgery, Vanderbilt University Medical Center, Nashville, TN 37203, USA; christina.e.bailey@vumc.org

**Keywords:** race, ethnic groups, neoplasms, healthcare disparities, mortality

## Abstract

**Simple Summary:**

The contribution of access to care to racial/ethnic disparities in cancer outcomes remains insufficiently investigated. Our study showed substantial racial/ethnic disparities in total mortality of the top five major cancers for U.S. men and women, across 4 major racial/ethnic populations. We found that the non-Hispanic (NH)-black and NH-white mortality disparity was most evident among patients with a high socioeconomic status (SES) or those who had private insurance, while the NH-white versus Hispanic and Asian disparities were more evident among patients with low SES, or those with no insurance or Medicare or Medicaid. Our findings highlight the need to develop racial/ethnic-specific strategies to reduce the disparity in mortality among cancer patients, and call for further investigation on factors other than access to care (e.g., lifestyle, treatment adherence, provider/patient relationships, etc.) for their contributions to the racial/ethnic disparities in cancer outcomes.

**Abstract:**

**Importance:** The reasons underlying racial/ethnic mortality disparities for cancer patients remain poorly understood, especially regarding the role of access to care. **Participants:** Over five million patients with a primary diagnosis of lung, breast, prostate, colon/rectum, pancreas, ovary, or liver cancer during 2004–2014, were identified from the National Cancer Database. Cox proportional hazards models were applied to estimate hazard ratios (HR) and 95% confidence intervals (CI) for total mortality associated with race/ethnicity, and access to care related factors (i.e., socioeconomic status [SES], insurance, treating facility, and residential type) for each cancer. **Results:** Racial/ethnic disparities in total mortality were observed across seven cancers. Compared with non-Hispanic (NH)-white patients, NH-black patients with breast (HR = 1.27, 95% CI: 1.26 to 1.29), ovarian (HR = 1.20, 95% CI: 1.17 to 1.23), prostate (HR = 1.31, 95% CI: 1.30 to 1.33), colorectal (HR = 1.11, 95% CI: 1.10 to 1.12) or pancreatic (HR = 1.03, 95% CI: 1.02 to 1.05) cancers had significantly elevated mortality, while Asians (13–31%) and Hispanics (13–19%) had lower mortality for all cancers. Racial/ethnic disparities were observed across all strata of access to care related factors and modified by those factors. NH-black and NH-white disparities were most evident among patients with high SES or those with private insurance, while Hispanic/Asian versus NH-white disparities were more evident among patients with low SES or those with no/poor insurance. **Conclusions and Relevance:** Racial/ethnic mortality disparities for major cancers exist across all patient groups with different access to care levels. The influence of SES or insurance on mortality disparity follows different patterns for racial/ethnic minorities versus NH-whites. **Impact:** Our study highlights the need for racial/ethnic-specific strategies to reduce the mortality disparities for major cancers.

## 1. Introduction

Cancer is the second leading cause of death in the United States (U.S.), accounting for over 22% of all deaths [1,2]. The top five leading causes of cancer death are cancers of the lung, prostate, colon/rectum, pancreas and liver among men, and cancers of the lung, breast, colon/rectum, pancreas, and ovary among women. These cancers account for over one-half of all cancer deaths in the U.S. [1]. Despite a substantial 27% decline in the U.S. cancer death rate over the past 25 years, improvement in cancer outcomes has not been equal across racial/ethnic groups [2].

The racial/ethnic disparity in cancer burden is a longstanding concern [3]. African Americans, the largest U.S. minority group, generally have higher mortality for most cancers than white individuals. Although the mortality gap has narrowed in the past decades for some cancers such as lung, colorectum, and prostate cancer [2,4], but it has remained for others [5]. This may result from the expanded access to high-quality cancer prevention and care, early detection, and advanced treatment in the US. [4] Data on cancer outcomes remain inconclusive for Hispanic and Asian Americans, although they are the two fastest-growing racial/ethnic groups in the U.S. [6,7].

Racial/ethnic disparities in cancer outcomes may reflect unequal access to care, resulting from social determinants (i.e., education/income levels, insurance, social status, and availability of quality services) [2]. In early 2019, the American Society of Clinical Oncology identified racial/ethnic disparities in access to care as a priority research area to accelerate the progress against cancer [8]. However, the contribution of access to care to racial/ethnic disparities in cancer outcomes was not well delineated, with conflicting results. Some studies suggest that black patients with similar medical care to white patients have similar cancer outcomes [9]. Others report that racial/ethnic disparities remain, even after accounting for factors related to access to care [10]. These inconsistencies may be due to the cancer type under study, sample size, study region, approach used to evaluate access to care, and covariates included for adjustment. Furthermore, few studies have investigated multiple cancers simultaneously or compared more than two racial/ethnic groups, which could help draw a bigger picture on this issue.

Herein, we used data from the National Cancer Database (NCDB) to comprehensively investigate race/ethnicity on mortality disparity in the five cancers with the highest mortality among U.S. men and women (seven cancers in total) and evaluate the influence of factors related to access to care and high-quality care on the disparity.

## 2. Methods

A cohort of 7,472,305 patients with a primary diagnosis of lung, breast, prostate, colorectal, pancreatic, ovarian, or liver cancers were identified from the NCDB (2004–2014), which captures over 70% of newly-diagnosed cancer cases in the U.S. [11]. Patients with a concomitant diagnosis or history of other malignancies (*n* = 1,233,424, 16.5%), whose TNM stages were inconsistent with those of site-specific metastasis (*n* = 165,113, 2.2%), and those with missing follow-up records (*n* = 548,484, 7.3%) or unknown race/ethnicity information (*n* = 390,003, 5.2%) were excluded. Because the NCDB data were completely de-identified, this study was approved as Exempt Human Research by the Institutional Review Board of Vanderbilt University; thus, no consent was needed.

Following the 2010 Census of Population and Housing [12], the NCDB defines race/ethnicity categories by a combination of race and Hispanic/Spanish origin. Patients recorded as “white” or “black”, and as “non-Hispanic/non-Spanish (NH)”, were categorized as “NH-white” and “NH-black”. Patients who reported to be of Hispanic or Spanish origin, or had a Spanish surname, were categorized as “Hispanic”, and patients of Asian origin were categorized as “Asian”. Patients recorded as “American Indian”, “Aleutian”, “Eskimo”, “Pacific Islander”, or other unspecified origin were excluded due to a relatively small sample size (i.e., accounting for 0.9% of patients under evaluation; Supplementary Table S1).

Demographic characteristics were obtained from the NCDB, including age at diagnosis, sex, year of diagnosis, rural/urban residence, annual household income and educational attainment, insurance type, treating facility, region, and distance to care. Residential type (“metro”, “urban”, or “rural”) was defined by matching patients’ Federal Information Processing Standards codes against U.S. Department of Agriculture Economic Research Service records from 2003 (for patients who were diagnosed with cancer before 2013) or 2013 (for those who were diagnosed with cancer in or after 2013). Income and educational attainment (percentage of adults who did not graduate high school) were estimated by matching patients’ resident zip codes against American Community Survey data from 2000 (for patients who were diagnosed with cancer before 2012) or 2012 (for those who were diagnosed with cancer in or after 2012), and both were categorized into quartiles in the NCDB [13]. Primary insurance at initial diagnoses was recorded as “not insured”, ‘private insurance”, “Medicaid”, “Medicare”, or “other government insurance”. Treating facility types were classified as ‘community cancer program”, ‘comprehensive community cancer program”, “academic/research program”, “integrated network cancer program”, or “other”, following the category classifications by the Commission on Cancer Accreditation program [13]. Facility locations were classified into “Northeast”, “Midwest”, “South”, and “West” in our analysis, following the U.S. Census Bureau definition [13]. Geographic distance from the facility was calculated in miles between patients’ resident zip code centroid and the case reporting facility’s street address [13].

Clinical and tumor characteristics were obtained from the NCDB and included histology type, grade, TNM stage, lymphovascular invasion, comorbidity (measured using Charlson/Deyo scores), and site-specific measurements (details in Supplementary Tables S2–S8). Information on receipt of primary cancer surgery, chemotherapy, endocrine therapy, radiotherapy, and immunotherapy (yes/no) was gathered from the NCDB. Intervals between diagnosis and treatment were obtained to account for the influence of delayed treatment initiation.

### Statistical Analysis

The primary outcome was overall survival (OS), defined as months from cancer diagnosis to death of any cause or to the last contact. To better assess the neighborhood-level socioeconomic status (SES), we created a single 3-level measure based on a combination of income and educational level quartiles. Patients within the highest quartiles of both income and education were categorized as high SES; patients within the lowest two quartiles of both income and education were categorized as low SES; otherwise, was categorized as intermediate SES.

Descriptive characteristics of different racial/ethnic groups were compared using chi-square tests for categorical variables and ANOVA for continuous variables. Five-year survival rates by race/ethnicity were estimated using the Kaplan–Meier method and compared by log-rank tests. Age, TNM stage, and treatment (i.e., surgery, chemotherapy, and radiotherapy) adjusted 5-year survival rates were estimated using stratified Cox models. Cox regression analyses were first conducted to derive hazard ratios (HR) and 95% confidence intervals (CI) for total mortality associated with race/ethnicity with adjustment for age, sex (if applicable), clinical characteristics, and treatment received. Stage-specific and sex-specific (for colorectal, pancreatic, liver, and lung cancer only) analyses were carried out to examine whether association patterns differed across stage or sex.

To evaluate the contributions of access to care related factors (i.e., SES, insurance, treating facility, facility location, and residence) on racial/ethnic disparities in cancer outcomes, we first estimated HRs and 95% CIs for overall mortality associated with the above factors for each cancer type using a multivariable Cox proportional hazard model with adjustment for race/ethnicity, age, sex (if applicable), clinical, and treatment characteristics. Second, we conducted stratified Cox regression analyses by access to care factors to investigate whether these factors modify racial/ethnic-mortality associations. Multiplicative interactions between race/ethnicity and the above stratified factors were evaluated using log likelihood ratio tests. Cancer-specific or disease-free survival were not evaluated, as death cause and cancer recurrence are not recorded in the NCDB. Alternatively, we conducted landmark analyses (i.e., 3-years post-diagnosis) to explore the nature of observed mortality differences. We evaluated the proportional hazard assumption by plotting scaled Schoenfeld residuals and potential collinearity for covariates in the Cox models using variable inflation factors (VIF). We found no evidence of assumption violation or collinearity. Missing rates for study variables were low (<3%); missing data were treated as a category for nominal variables or replaced with overall (distance-to-care) or stage-specific median values (interval from diagnosis to treatment) for continuous variables. We also conducted sensitivity analyses by excluding patients with missing information on access to care variables to evaluate the robustness of our analyses.

All statistical tests were based on 2-sided probability with significance levels set at *p * < 0.05, with no adjustment for multiple comparisons. All statistical analyses were performed using R 3.5.1 (R Foundation, Vienna, Austria).

## 3. Results

A total of 5,077,413 patients with a diagnosis of breast, ovarian, prostate, colorectal, pancreatic, liver, or lung cancer were included in the final analysis (Supplementary Table S1). Overall, compared to NH-white patients, NH-black, Hispanic, and Asian patients were more likely to be young, uninsured, or insured by Medicaid, and reside in metropolitan areas; more NH-black and Hispanic patients had low SES, while Asian patients were more likely to have high SES. Private insurance was more frequently reported among Asian compared with NH-white, NH-black, and Hispanic patients. The majority of NH-black patients were from the southern region; two-thirds of Hispanic patients were from southern and western regions, while approximately one-half of Asian patients were from the western region (Supplementary Tables S2–S8).

Overall, for all cancers except pancreatic cancer, Asian patients showed the highest 5-year survival rate, followed by Hispanic and NH-white groups, and NH-black patients had the lowest survival rates. For pancreatic cancer, NH-white patients had a slightly lower survival rate than NH-black patients (9.6% vs. 10.0%, respectively), and both were significantly lower than Hispanic and Asian patients (14.1% vs.14.0%, respectively; Table 1 and Supplementary Figures S1 and S2). Age, TNM stage, and treatments adjusted 5-year survival rates showed similar patterns of racial/ethnic differences (Table 1).

The racial/ethnic disparities in total mortality remained after adjustment for age, sex (if applicable), clinical factors, and treatment received (Table 2). Compared with NH-white, NH-black patients had significantly elevated mortality risk for cancers of the breast (HR = 1.27, 95% CI: 1.26 to 1.29), ovary (HR = 1.20, 95% CI: 1.17 to 1.23), prostate (HR = 1.31, 95% CI: 1.30 to 1.33), colon/rectum (HR = 1.11, 95% CI: 1.10 to 1.12), and pancreas (HR = 1.03, 95% CI: 1.02 to 1.05) but comparable or slightly lower risk for cancers of the liver (HR = 1.00, 95% CI: 0.98 to 1.02) and lung (HR = 0.98, 95% CI: 0.97 to 0.98). Asian and Hispanic patients had significantly (16−31%) lower mortality risk across all seven cancer types compared with NH-white patients. The disparity patterns were consistent across TNM stages for all seven cancers (Supplementary Table S9) and did not differ by sex for colorectal, pancreatic, liver, and lung cancers (Supplementary Table S10).

Factors related to access to care were significantly associated with total mortality (Table 3). Patients with high neighborhood-level SES, private insurance, who were treated in academic/research facilities, or from the Northeastern region had significantly lower mortality risk than their counterparts across all cancer types.

**Figure 1:**
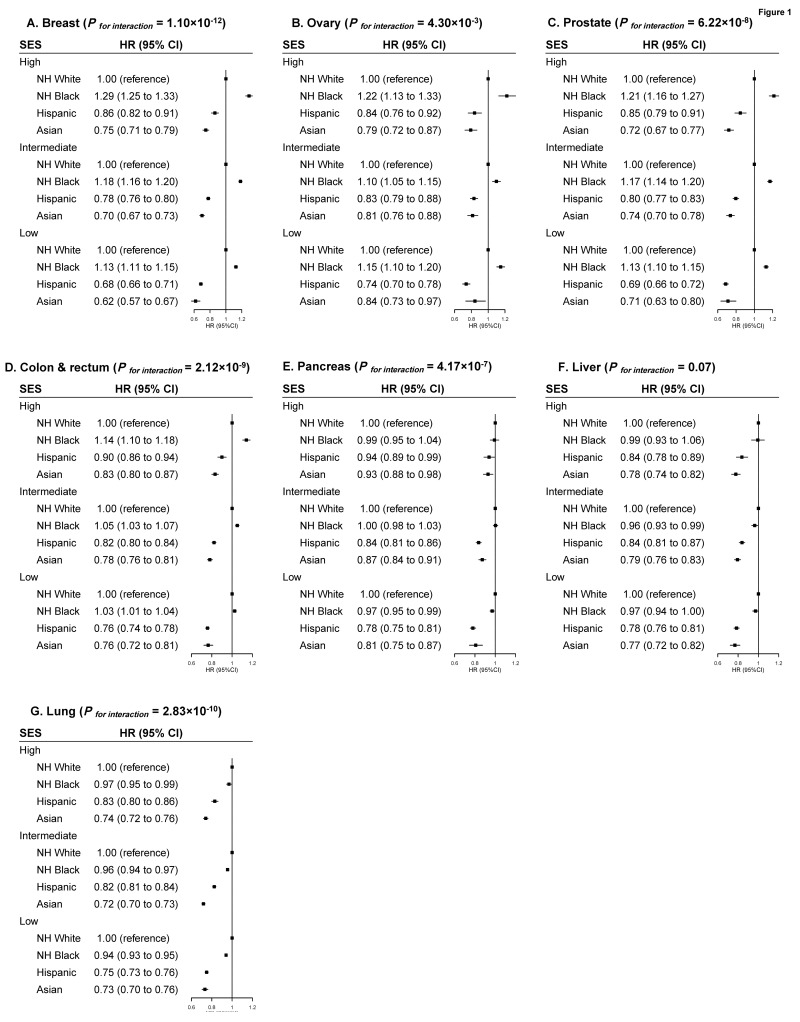
Multivariable-adjusted HRs and 95% CIs for Total Mortality Associated with Race/ethnicity According to Socioeconomic Status (SES) in Major Cancer Types ((**A**) Breast Cancer; (**B**) Ovarian Cancer; (**C**) Prostate Cancer; (**D**) Colorectal Cancer; (**E**) Pancreatic Cancer; (**F**) Liver Cancer; (**G**) Lung Cancer). The HRs and 95% CIs for total mortality associated with race/ethnicity according to Socioeconomic Status (SES) in major cancer types. The HRs and 95% CIs were adjusted for age, sex (if applicable), biology (histology type, grade, TNM stage, LVI, comorbidity; ER, PR, HER2 for breast cancer; CA 125 for ovarian cancer; PSA and Gleason grade for prostate cancer; CA 19-9 for pancreatic cancer; AFP, Fibrosis Score, INR for liver cancer; separate tumor nodules for lung cancer; primary site, CEA, circumferential resection margin for colorectal cancer), treatment factors (surgery, chemotherapy, interval between diagnosis to first treatment; and endocrine therapy, radiation, immunotherapy, if applicable) and access to care (insurance, facility type, region, urban/rural residence, distance to care, and year of diagnosis). *p* values for interaction between racial/ethnic groups and SES groups were derived from log likelihood tests. Abbreviation: HR, Hazard Ratio; CI, Confidence Interval.

**Figure 2:**
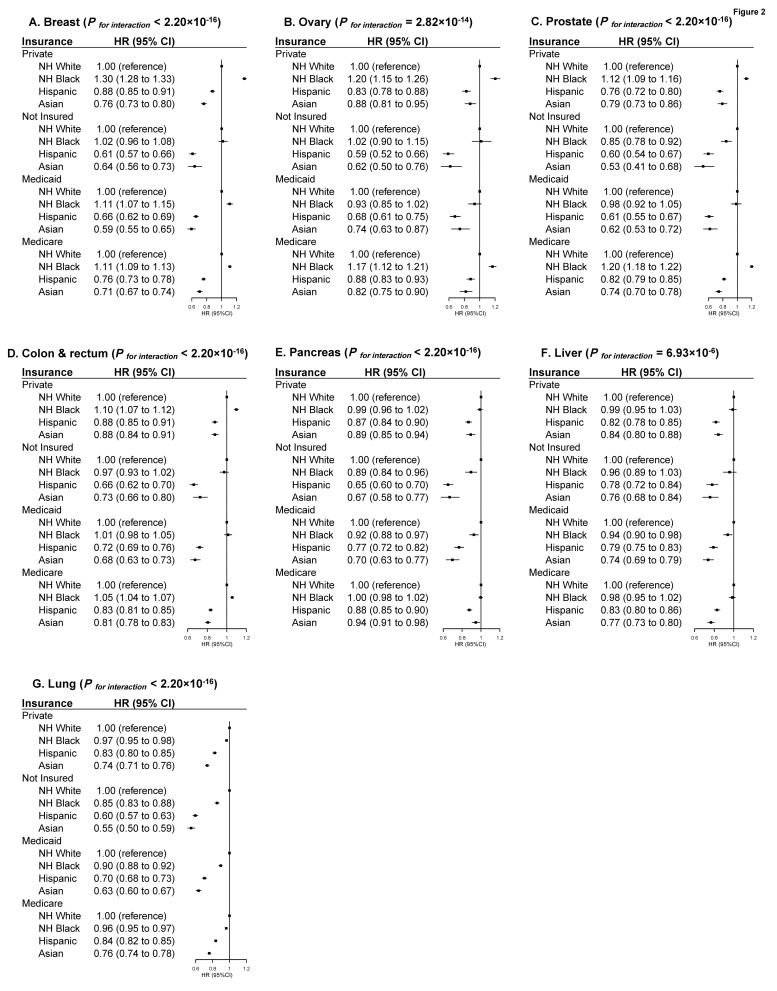
Multivariable-adjusted HRs and 95% CIs for Total Mortality Associated with Race/ethnicity According to Insurance Status in Major Cancer Types. The HRs and 95% CIs for total mortality associated with race/ethnicity according to insurance status in major cancer types ((**A**) Breast Cancer; (**B**) Ovarian Cancer; (**C**) Prostate Cancer; (**D**) Colorectal Cancer; (**E**) Pancreatic Cancer; (**F**) Liver Cancer; (**G**) Lung Cancer). The HRs and 95% CIs were adjusted for age, sex (if applicable), biology (histology type, grade, TNM stage, LVI, comorbidity; ER, PR, HER2 for breast cancer; CA 125 for ovarian cancer; PSA and Gleason grade for prostate cancer; CA 19-9 for pancreatic cancer; AFP, Fibrosis Score, INR for liver cancer; separate tumor nodules for lung cancer; primary site, CEA, circumferential resection margin for colorectal cancer), treatment factors (surgery, chemotherapy, interval between diagnosis to first treatment; and endocrine therapy, radiation, immunotherapy, if applicable) and access to care (education, income, facility type, region, urban/rural residence, distance to care, and year of diagnosis). *p* values for interaction between racial/ethnic groups and insurance groups were derived from log likelihood test. Cancer patients with unspecified government insurance or unknown insurance status were not included in this analysis. Abbreviation: HR, Hazard Ratio; CI, Confidence Interval.

**Figure 3:**
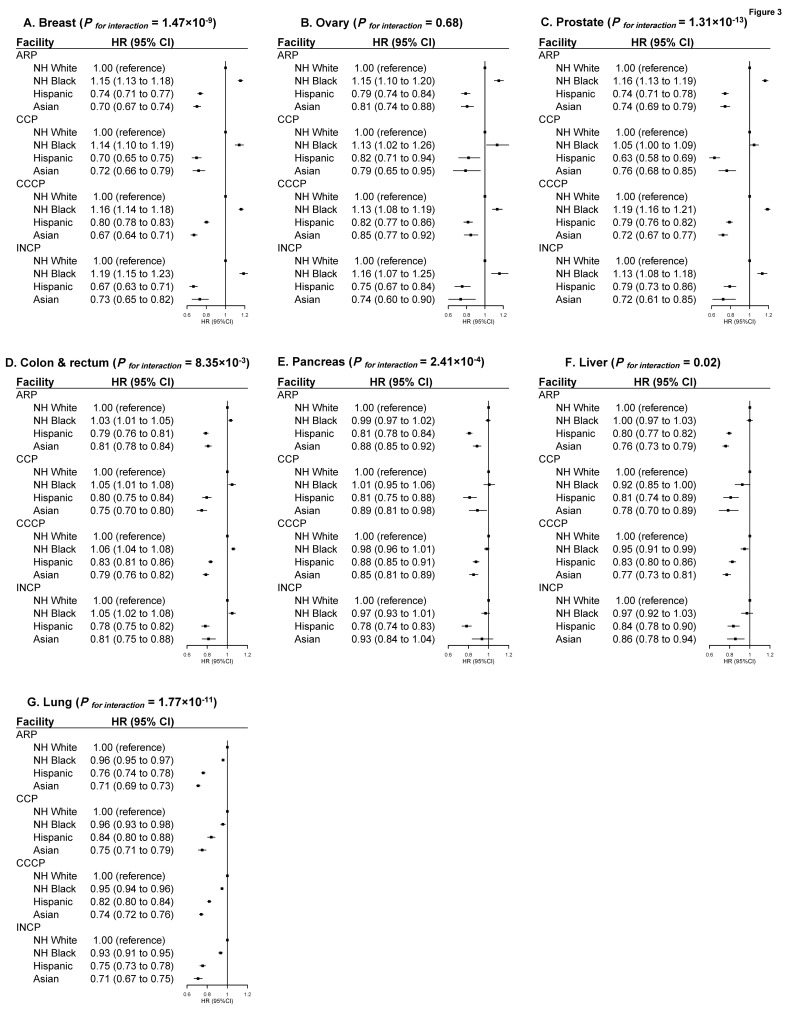
Multivariable-adjusted HRs and 95% CIs for Total Mortality Associated with Race/ethnicity According to Treating Facility Type in Major Cancer Types. The HRs and 95% CIs for total mortality associated with race/ethnicity according to treatment facility type in major cancer types ((**A**) Breast Cancer; (**B**) Ovarian Cancer; (**C**) Prostate Cancer; (**D**) Colorectal Cancer; (**E**) Pancreatic Cancer; (**F**) Liver Cancer; (**G**) Lung Cancer). The HRs and 95% CIs were adjusted for age, sex (if applicable), biology (histology type, grade, TNM stage, LVI, comorbidity; ER, PR, HER2 for breast cancer; CA 125 for ovarian cancer; PSA and Gleason grade for prostate cancer; CA 19-9 for pancreatic cancer; AFP, Fibrosis Score, INR for liver cancer; separate tumor nodules for lung cancer; primary site, CEA, circumferential resection margin for colorectal cancer), treatment factors (surgery, chemotherapy, interval between diagnosis to first treatment; and endocrine therapy, radiation, immunotherapy, if applicable) and access to care (education, income, insurance, region, urban/rural residence, distance to care, and year of diagnosis). *p* values for interaction between racial/ethnic groups and facility groups were derived from log likelihood tests. Cancer patients with unknown treatment facility type were not included in this analysis. Abbreviation: HR, Hazard Ratio; CI, Confidence Interval.

Significant interactions between neighborhood-level SES and race/ethnicity were observed for all cancers except liver cancer, although the pattern was similar for all cancers. For NH-black patients with breast, ovarian, prostate or colorectal cancers, compared with NH-white counterparts, excess mortality was most evident among patients with high SES (14% to 29%, respectively), but smaller for patients with low SES (3% to 15%, respectively). On the other hand, the lower mortality observed for Hispanic and Asian versus NH-white patients was more evident among patients with low SES than those with high SES (Figure 1). When stratified by insurance type, the white-black disparity in mortality after diagnosis of breast, ovarian, prostate, or colorectal cancers was most evident among patients with private insurance (10% to 30%, respectively), while little disparity was observed among those with Medicaid or no insurance. The Hispanic and Asian versus NH-white mortality differences were more evident among patients with no insurance than those with private insurance (Figure 2). No clear patterns were observed regarding the variation of racial/ethnic mortality disparities by treating facility, region, and residence (Figure 3 and Supplementary Figures S3 and S4), although the *p* values were significant for many of the multiplicative interaction tests.

These patterns were very similar for 3-year mortality (Supplementary Tables S11–S15). Sensitivity analyses excluding individuals with missing information on access to care variables, total exclusion ranging from 6.0% to 11.7% of entire populations across cancer sites, showed similar association patterns between racial/ethnic groups and overall mortality for all cancers under study (Supplementary Table S16).

## 4. Discussion

In this large-scale registry-based study, we found a substantial racial/ethnic disparity in total mortality across seven major cancers in U.S. men and women. Of the four racial/ethnic groups evaluated in this study, NH-black patients experienced the highest mortality after cancer diagnosis. Hispanic and Asian patients, however, showed the lowest mortality post-cancer diagnosis. These racial/ethnic disparities presented across all patient subgroups with different access to care characteristics. NH-black and NH-white mortality differences were most evident among patients with high SES or private insurance, while Hispanic and Asian versus NH-white mortality differences were more evident among patients with low SES.

It has been suggested that racial/ethnic disparities in cancer outcomes might largely reflect disproportionate poverty levels [2] and predominantly arise from unequal access to high-quality care [2,3,6,14]. In our study, higher proportions of NH-black and Hispanic patients were in the lowest quartiles of income or educational attainment, and were uninsured or poorly insured compared to NH-white or Asian patients, consistent with U.S. Census Bureau data [15]. Poverty or lack of insurance influences access to appropriate preventive care, cancer screening or early diagnosis, and receiving optimal treatment, which are closely related to cancer mortality [16,17]. A prior study reported that people with lower SES or fewer years of education had higher mortality after cancer diagnosis than their counterparts, regardless of race/ethnicity [18,19]. Furthermore, studies found that the disparity between NH-black and NH-white cancer patients related to access-to-timely-treatment was nearly eliminated with Medicaid Expansion [20,21]. Consistent with these studies, we found that patients with better access to care and high-quality care (i.e., higher SES, better insurance, metropolitan residence, treatment in academic/research facilities) had significantly lower mortality across all cancers and all racial/ethnic populations than their counterparts with worse access to care.

However, our study does not support that the racial/ethnic mortality disparity is fully explained by unequal access to care. In contrast, we found that mortality disparity between NH-black versus NH-white patients with breast, ovarian, prostate, and colorectal cancers widened among patients with high SES or private insurance, compared to low SES or uninsured patients. In line with our findings, an earlier study also showed that death risk for NH-black compared to NH-white patients increased with higher neighborhood SES [18]. It was proposed that racial/ethnic minorities do not achieve as much gain in health from improved socioeconomic situations as their NH-white counterparts, known as the “diminishing returns” hypothesis [22,23]. Our finding that the survival benefit narrowed among Hispanic and Asian versus NH-white patients with high SES or private insurance, however, appears to contradict this hypothesis. These findings call for more in-depth research on SES-related factors that may influence cancer outcomes, e.g., screening practice, treatment compliance and post-cancer care. Cultural heterogeneity, such as lifestyle (e.g., dietary habits, physical activity, and obesity), traditional/alternative medicine use (acupuncture and yoga), attitudes or beliefs, and psychosocial support can lead to different health outcomes and should be taken into consideration in cancer care continuum to help reduce the mortality disparity [24]. In addition, studies have shown that the quality of patient-provider communication varies by race/ethnicity [25,26]. Health providers’ implicit racial biases were negatively associated with oncologists’ communication, as well as patients’ perception of recommended treatment [27], which may also contribute to the racial/ethnic disparities.

In our study, Asian patients had a substantial survival advantage over NH-whites for all cancers evaluated. This may suggest that underlying genetic/biological differences, as indicated by studies on the mutational landscape in multiple cancers [28,29,30], may play a role [31]. Unfortunately, these factors could not be evaluated in our study due to unavailable information. However, the influence of culture, lifestyle and social support, and their interactions with treatment and genetic factors on mortality disparity, should not be overlooked.

It is noteworthy that racial/ethnic disparities exist in all patient subgroups with different levels of access to care. This finding is consistent with data from the California Cancer Registry, in which neighborhood SES explained only 5–7% of mortality disparities between NH-white, NH-black, and Hispanic patients with breast, prostate, and colorectal cancers, and 18% with lung cancer [32]. Studies have also shown that disparities remained in cancer-specific and all-cause mortality between white and black breast or prostate cancer patients after accounting for SES/access to care and biological differences [33,34]. Our observation is also consistent with a report from the Southwestern Oncology Group, which shows that in clinical-trial settings with uniform disease stage and treatment, African Americans with sex-specific cancers had worse survival than white patients, even after an additional adjustment for SES [10].

Higher mortality has been previously reported for almost all cancers among NH-black patients than NH-white counterparts [5,35,36,37]. However, in our study, NH-black patients with pancreatic, liver, or lung cancers had slightly lower mortality than NH-white patients. Our findings are consistent with prior studies that have taken biology and treatment into account [10,18]. This may be due to the influence of aggressive biologic characteristics, which may outweigh race/ethnicity or access to care for fatal cancers (e.g., pancreatic, liver, and lung cancers) [9]. Younger age at diagnosis, and thus, better physical fitness and higher tolerance to aggressive treatments, may be another possible explanation for this disparity between NH-black compared to NH-white patients.

The mortality disparity after cancer diagnosis has been controversial for Hispanic compared with NH-white patients. Based on data from Surveillance, Epidemiology, and End Results (SEER) Program, the American Cancer Society reported similar 5-year survival between Hispanic and NH-white patients for almost all cancers [6], while several other studies have shown that, after adjustment for age, stage, and sex (for non-sex-specific cancers), Hispanic patients have a higher mortality from most cancers, with lung cancer being an exception [5,35,36]. In contrast, we found that Hispanic patients had better survival than NH-white patients across nearly all seven cancers examined, and this disparity remained after adjustment for age, clinical factors, treatment, and factors related to access to care. In comparison to the SEER program, which covers approximately 35% of the U.S. population, the NCDB represents more than 70% of newly diagnosed cancer cases nationwide, and thus, may have better generalizability. Nonetheless, the incomplete death ascertainment due to return migration after a cancer diagnosis, and the selectively healthy immigrant population, may also be a possible explanation for our observed favorable outcome [38,39,40]. However, in our study, although the median follow-up periods of Hispanic patients were shorter than those of NH-white patients for some cancers (e.g., breast, prostate, colorectal cancer), they were longer than those of NH-black patients for all cancers. In addition, lower mortality was seen across tumor stages and insurance types and persisted in both western and southern regions, where Hispanic populations differed. These findings call for research on underlying contributors, particularly regarding non-clinical attributes such as lifestyle factors and social support, to the survival disparity in cancer patients.

The strengths of our study include the large number of patients and detailed information on clinical and treatment factors, which enabled us to account for a wide range of covariates and conduct subgroup analyses. We systemically evaluated the top five major cancer deaths in U.S. men and women in four major racial/ethnic categories. Using the NCDB, we included approximately 70% of eligible cancer patients diagnosed between 2000 and 2014 across the U.S., reflecting contemporary diagnostic and treatment patterns and ensuring high generalizability. There are also some limitations of our study. First, we employed neighborhood-level, rather than individual-level, education and income as a measure of SES in our study, so misclassification cannot be ruled out. Second, cancer-specific mortality and recurrence were not assessed in our study due to unavailable information. However, we carried out 3-year survival analyses and found similar disparity patterns. Third, each racial/ethnic group evaluated in this study was comprised of numerous subgroups with heterogeneous cultural and socioeconomic backgrounds; thus, the aggregated racial/ethnic group may mask the substantial heterogeneity by origin [41,42]. Furthermore, as a registry-based study, potential misclassifications of racial/ethnic categories in medical records and selection bias cannot be ruled out, particularly given that 13.5% of patients were excluded from the study due to a lack of information on race/ethnicity and follow-up, which may differ from those included in our current study on factors related to access to care [43]. Finally, information was unavailable on other socioeconomic determinants which may also mediate access to care (e.g., treatment adherence, provider/patient relationships, social support).

## 5. Conclusions

In conclusion, racial/ethnic disparities in mortality for the top seven major cancers in the U.S. exist across all populations with different access to care, even after an adjustment for a wide range of prognostic factors. Furthermore, NH-black and NH-white disparities widened, while disparities between NH-white, Hispanic, and Asian patients narrowed among patients with high SES or private insurance. While more research is needed to understand the underlying mechanisms for the different patterns of disparities across racial/ethnic groups, our findings indicate racial/ethnic-specific strategies are needed to deploy effective and targeted preventive measures.

## Figures and Tables

**Table 1:** Five-year survival rates of major cancer types according to race/ethnicity.

Cancer Site	Unadjusted 5-Year Survival Rates (%)				Adjusted 5-Year Survival Rates (%) *			
	NH-White	NH-Black	Hispanic	Asian	NH-White	NH-Black	Hispanic	Asian
**Breast**	86.9	80.8	89.0	92.6	91.4	87.4	92.0	93.7
**Ovary**	46.7	40.4	56.3	62.4	49.7	44.4	56.1	56.8
**Prostate**	88.8	85.8	89.1	90.6	92.4	89.3	93.1	94.6
**Colon and Rectum**	58.1	55.1	63.4	66.3	61.3	56.9	64.5	67.0
**Pancreas**	9.6	10.0	14.1	14.0	3.7	4.8	8.0	7.0
**Liver**	20.9	18.0	23.6	31.1	13.4	13.7	18.7	21.7
**Lung**	19.7	17.9	23.0	26.0	11.5	12.5	19.5	22.2

* These survival rates were adjusted for age, TNM stage, and receipt of treatment (i.e., surgery, chemotherapy, radiotherapy).

**Table 2:** Association of race/ethnicity with total mortality with adjustment for clinical characteristics.

Race/Ethnicity	HR (95% CI) *						
	Breast Cancer	Ovarian Cancer	Prostate Cancer	Colorectal Cancer	Pancreatic Cancer	Liver Cancer	Lung Cancer
**NH-white**	1.00 (reference)	1.00 (reference)	1.00 (reference)	1.00 (reference)	1.00 (reference)	1.00 (reference)	1.00 (reference)
**NH-black**	1.27 (1.26 to 1.29)	1.20 (1.17 to 1.23)	1.31 (1.30 to 1.33)	1.11 (1.10 to 1.12)	1.03 (1.02 to 1.05)	1.00 (0.98 to 1.02)	0.98 (0.97 to 0.98)
**Hispanic**	0.84 (0.83 to 0.86)	0.84 (0.81 to 0.87)	0.84 (0.82 to 0.86)	0.87 (0.85 to 0.88)	0.86 (0.84 to 0.88)	0.82 (0.81 to 0.84)	0.81 (0.80 to 0.82)
**Asian**	0.69 (0.67 to 0.71)	0.80 (0.76 to 0.85)	0.70 (0.67 to 0.73)	0.79 (0.78 to 0.81)	0.87 (0.85 to 0.90)	0.78 (0.76 to 0.80)	0.72 (0.71 to 0.73)

Abbreviation: HR, hazard ratio; CI, confidence interval. * Adjusted for age, sex (if applicable), biological (histology type, grade, TNM stage, LVI, comorbidity; ER, PR, HER2 for breast cancer; CA 125 for ovarian cancer; PSA and Gleason grade for prostate cancer; CA 19-9 for pancreatic cancer; AFP, Fibrosis Score, INR for liver cancer; separate tumor nodules for lung cancer; primary site, CEA, circumferential resection margin for colorectal cancer), treatment factors (surgery, chemotherapy, interval between diagnosis to first treatment; endocrine therapy, radiation, immunotherapy, if applicable) and year of diagnosis.

**Table 3:** Association of factors related to access to care with total mortality.

				HR (95% CI)			
	Breast Cancer	Ovarian Cancer	Prostate Cancer	Colorectal Cancer	Pancreatic Cancer	Liver Cancer	Lung Cancer
**Neighborhood-level SES**							
Low	1.00 (reference)	1.00 (reference)	1.00 (reference)	1.00 (reference)	1.00 (reference)	1.00 (reference)	1.00 (reference)
Intermediate	0.95 (0.94 to 0.96)	0.93 (0.91 to 0.95)	0.86 (0.85 to 0.88)	0.95 (0.94 to 0.96)	0.96 (0.94 to 0.97)	0.98 (0.96 to 0.99)	0.97 (0.96 to 0.97)
High	0.82 (0.81 to 0.83)	0.84 (0.82 to 0.86)	0.71 (0.70 to 0.72)	0.86 (0.85 to 0.87)	0.89 (0.88 to 0.90)	0.91 (0.89 to 0.93)	0.91 (0.90 to 0.92)
**Insurance**							
Private	1.00 (reference)	1.00 (reference)	1.00 (reference)	1.00 (reference)	1.00 (reference)	1.00 (reference)	1.00 (reference)
Not insured	1.63 (1.59 to 1.67)	1.21 (1.17 to 1.27)	1.68 (1.62 to 1.74)	1.44 (1.42 to 1.47)	1.15 (1.12 to 1.18)	1.24 (1.20 to 1.28)	1.24 (1.23 to 1.26)
Medicaid	1.77 (1.74 to 1.79)	1.32 (1.27 to 1.37)	1.83 (1.78 to 1.89)	1.50 (1.48 to 1.53)	1.20 (1.18 to 1.23)	1.19 (1.16 to 1.21)	1.22 (1.21 to 1.23)
Medicare	1.23 (1.22 to 1.25)	1.06 (1.04 to 1.08)	1.18 (1.16 to 1.19)	1.14 (1.13 to 1.15)	1.07 (1.05 to 1.08)	1.12 (1.10 to 1.14)	1.10 (1.10 to 1.11)
Other government	1.17 (1.11 to 1.22)	1.14 (1.05 to 1.25)	1.38 (1.33 to 1.44)	1.11 (1.07 to 1.15)	1.06 (1.01 to 1.10)	1.09 (1.03 to 1.15)	1.05 (1.03 to 1.07)
Unknown	1.05 (1.02 to 1.08)	0.95 (0.90 to 1.01)	1.00 (0.97 to 1.04)	1.10 (1.07 to 1.13)	0.97 (0.94 to 1.00)	1.09 (1.04 to 1.14)	1.07 (1.05 to 1.08)
**Urban/rural Residence**							
Metro	1.00 (reference)	1.00 (reference)	1.00 (reference)	1.00 (reference)	1.00 (reference)	1.00 (reference)	1.00 (reference)
Urban	1.03 (1.01 to 1.04)	0.99 (0.96 to 1.01)	1.01 (0.99 to 1.03)	1.02 (1.01 to 1.03)	1.03 (1.02 to 1.05)	1.10 (1.07 to 1.12)	1.01 (1.01 to 1.02)
Rural	1.02 (0.99 to 1.05)	1.02 (0.96 to 1.08)	0.97 (0.94 to 1.01)	0.99 (0.96 to 1.01)	1.02 (0.99 to 1.06)	1.07 (1.01 to 1.14)	1.01 (1.00 to 1.03)
Unknown	1.32 (1.29 to 1.35)	1.26 (1.21 to 1.31)	1.32 (1.28 to 1.36)	1.27 (1.24 to 1.29)	1.12 (1.09 to 1.14)	1.20 (1.16 to 1.24)	1.13 (1.12 to 1.15)
**Facility type**							
Community	1.00 (reference)	1.00 (reference)	1.00 (reference)	1.00 (reference)	1.00 (reference)	1.00 (reference)	1.00 (reference)
Comprehensive Community	0.95 (0.94 to 0.96)	1.00 (0.97 to 1.03)	0.90 (0.89 to 0.92)	0.98 (0.97 to 0.99)	0.96 (0.94 to 0.97)	0.95 (0.92 to 0.98)	0.97 (0.96 to 0.98)
Academic/Research	0.85 (0.83 to 0.86)	0.95 (0.92 to 0.98)	0.76 (0.75 to 0.77)	0.90 (0.89 to 0.91)	0.81 (0.80 to 0.83)	0.77 (0.74 to 0.79)	0.89 (0.89 to 0.90)
Integrated Network	0.91 (0.89 to 0.92)	1.00 (0.97 to 1.04)	0.86 (0.84 to 0.88)	0.96 (0.95 to 0.98)	0.92 (0.90 to 0.94)	0.91 (0.88 to 0.95)	0.95 (0.94 to 0.95)
Unknown	2.34 (2.28 to 2.41)	1.06 (0.99 to 1.12)	5.01 (3.92 to 6.41)	2.02 (1.96 to 2.07)	0.81 (0.77 to 0.85)	0.86 (0.80 to 0.92)	0.92 (0.89 to 0.95)
**Region**							
Northeast	1.00 (reference)	1.00 (reference)	1.00 (reference)	1.00 (reference)	1.00 (reference)	1.00 (reference)	1.00 (reference)
Midwest	1.06 (1.05 to 1.08)	1.07 (1.05 to 1.10)	1.04 (1.03 to 1.06)	1.06 (1.05 to 1.07)	1.12 (1.10 to 1.13)	1.12 (1.09 to 1.14)	1.08 (1.08 to 1.09)
South	1.01 (0.99 to 1.02)	0.98 (0.96 to 1.00)	1.04 (1.02 to 1.05)	1.06 (1.05 to 1.07)	1.10 (1.09 to 1.11)	1.07 (1.05 to 1.09)	1.04 (1.03 to 1.05)
West	0.94 (0.93 to 0.96)	1.00 (0.97 to 1.03)	0.95 (0.93 to 0.96)	1.03 (1.02 to 1.05)	1.07 (1.05 to 1.08)	1.07 (1.05 to 1.10)	1.06 (1.05 to 1.06)

Abbreviation: HR, hazard ratio; CI, confidence interval; SES, socioeconomic status. Adjusted for age, sex (if applicable), race/ethnicity, biological (histology type, grade, TNM stage, LVI, comorbidity; ER, PR, HER2 for breast cancer; CA 125 for ovarian cancer; PSA and Gleason grade for prostate cancer; CA 19-9 for pancreatic cancer; AFP, Fibrosis Score, INR for liver cancer; separate tumor nodules for lung cancer; primary site, CEA, circumferential resection margin for colorectal cancer), treatment factors (surgery, chemotherapy, interval between diagnosis to first treatment; endocrine therapy, radiation, immunotherapy, if applicable) and access to care (neighborhood-level SES, urban/rural residence, insurance, facility type, distance to care, region, year of diagnosis).

## Data Availability

The analytical data were obtained from the American College of Surgeons’ National Cancer Database. Information on accessing the data is available at https://www.facs.org/quality-programs/cancer/ncdb/publicaccess. The analytical codes related to the current project are available upon reasonable request (corresponding to: xiao-ou.shu@vanderbilt.edu).

## Supplementary Materials

The following supporting information can be downloaded at: https://www.mdpi.com/article/10.3390/cancers14143390/s1, Supplementary Table S1. Number of Cases by Cancer Sites and Race/ethnicity; Supplementary Table S2. Characteristics of Female Breast Cancer by Race/ethnicity; Supplementary Table S3. Characteristics of Ovarian Cancer by Race/ethnicity; Supplementary Table S4. Characteristics of Prostate Cancer by Race/ethnicity; Supplementary Table S5. Characteristics of Colorectal Cancer by Race/ethnicity; Supplementary Table S6. Characteristics of Pancreatic Cancer by Race/ethnicity; Supplementary Table S7. Characteristics of Liver Cancer by Race/ethnicity; Supplementary Table S8. Characteristics of Lung Cancer by Race/ethnicity; Supplementary Table S9. Multivariable-adjusted HRs and 95% CIs for Total Mortality in Association with Race/ethnicity of Major Cancer Types According to Stage; Supplementary Table S10. Multivariable-adjusted HRs and 95% CIs for Total Mortality of Major Cancer Types According to SEX; Supplementary Table S11. Multivariable-adjusted HRs and 95% CIs for 3-year Mortality Associated with Race/ethnicity According to Socioeconomic Status (SES) in Major Cancer Types; Supplementary Table S12. Multivariable-adjusted HRs and 95% CIs for 3-year Mortality Associated with Race/ethnicity According to Insurance Status in Major Cancer Types; Supplementary Table S13. Multivariable-adjusted Hazard Ratio (HR) and 95% CIs for 3-year Mortality Associated with Race/ethnicity According to Treating Facility Type in Major Cancer Types; Supplementary Table S14. Multivariable-adjusted HRs and 95% CIs for 3-year Mortality Associated with Race/ethnicity According to Region in Major Cancer Types; Supplementary Table S15. Multivariable-adjusted HRs and 95% CIs for 3-year Mortality Associated with Race/ethnicity According to Urban/rural Residence in Major Cancer Types; Supplementary Table S16. HRs and 95% CIs for Total Mortality Associated with Race/ethnicity (sensitivity analysis excluding individuals with missing information on access-to-care related factors); Supplementary Figure S1. Crude Overall Survival of Sex-specific Cancers; Supplementary Figure S2. Crude Overall Survival of Lung, Liver, Pancreatic and Colorectal Cancers by Sex; Supplementary Figure S3. The multivariable-adjusted HRs and 95% CIs for total mortality associated with race/ethnicity according to region in major cancer types; Supplementary Figure S4. The multivariable-adjusted HRs and 95% CIs for total mortality associated with race/ethnicity according to urban/rural residence in major cancer types.
