# Supplementary material for: Racial/Ethnic Disparities in Mortality Related to Access to Care for Major Cancers in the United States
Source: Cancers (Basel). 2022 Jul 12;14(14):3390. doi: 10.3390/cancers14143390 (PMC9318931; doi:10.3390/cancers14143390)
Supplement: Supplementary file 1 [file cancers-14-03390-s001.zip › cancers-1789753-supplementary.pdf]

Online-Only Supplements

A total of 16 online-only tables and 4 online-only figures are included in this document.

Supplementary Table S1. Number of Cases by Cancer Sites and Race/ethnicity

Supplementary Table S2. Characteristics of Female Breast Cancer by Race/ethnicity

Supplementary Table S3. Characteristics of Ovarian Cancer by Race/ethnicity

Supplementary Table S4. Characteristics of Prostate Cancer by Race/ethnicity

Supplementary Table S5. Characteristics of Colorectal Cancer by Race/ethnicity

Supplementary Table S6. Characteristics of Pancreatic Cancer by Race/ethnicity

Supplementary Table S7. Characteristics of Liver Cancer by Race/ethnicity

Supplementary Table S8. Characteristics of Lung Cancer by Race/ethnicity

Supplementary Table S9. Multivariable-adjusted HRs and 95% CIs for Total Mortality in Association with Race/ethnicity of Major Cancer Types According to Stage

Supplementary Table S10. Multivariable-adjusted HRs and 95% CIs for Total Mortality of Major Cancer Types According to SEX

Supplementary Table S11. Multivariable-adjusted HRs and 95% CIs for 3-year Mortality Associated with Race/ethnicity According to Socioeconomic Status (SES) in Major Cancer Types

Supplementary Table S12. Multivariable-adjusted HRs and 95% CIs for 3-year Mortality Associated with Race/ethnicity According to Insurance Status in Major Cancer Types

Supplementary Table S13. Multivariable-adjusted Hazard Ratio (HR) and 95% CIs for 3-year Mortality Associated with Race/ethnicity According to Treating Facility Type in Major Cancer Types

Supplementary Table S14. Multivariable-adjusted HRs and 95% CIs for 3-year Mortality Associated with Race/ethnicity According to Region in Major Cancer Types

Supplementary Table S15. Multivariable-adjusted HRs and 95% CIs for 3-year Mortality Associated with Race/ethnicity According to Urban/rural Residence in Major Cancer Types

Supplementary Table S16. HRs and 95% CIs for Total Mortality Associated with Race/ethnicity (sensitivity analysis excluding individuals with missing information on access-to-care related factors)

Supplementary Figure S1. Crude Overall Survival of Sex-specific Cancers

Supplementary Figure S2. Crude Overall Survival of Lung, Liver, Pancreatic and Colorectal Cancers by Sex

Supplementary Figure S3. The multivariable-adjusted HRs and 95% CIs for total mortality associated with race/ethnicity according to region in major cancer types.

Supplementary Figure S4. The multivariable-adjusted HRs and 95% CIs for total mortality associated with race/ethnicity according to urban/rural residence in major cancer types.

Supplementary Table S1. Number of Cases by Cancer Sites and Race/ethnicity.

Cancer Site	NH-White, N (%)		NH-Black, N (%)		Hispanic, N (%)		Asian, N (%)		Sum, N
**Breast Cancer**	**1,322,385**	**(79.4)**	**193,481**	(11.6)	94,360	(5.7)	54,271	(3.3)	1,664,497
**Ovarian Cancer**	108,578	(82.0)	11,155	(8.4)	8,474	(6.4)	4,204	(3.2)	132,411
**Prostate Cancer**	826,152	(79.1)	152,571	(14.6)	47,796	(4.6)	18,563	(1.8)	1,045,082
**Colorectal Cancer**	635,569	(78.8)	100,124	(12.4)	46,270	(5.7)	24,413	(3.0)	806,376
**Pancreatic Cancer**	182,538	(79.2)	28,935	(12.6)	12,920	(5.6)	5,970	(2.6)	230,363
**Liver Cancer**	74,992	(63.5)	18,299	(15.5)	15,455	(13.1)	9,273	(7.9)	118,019
**Lung Cancer**	908,291	(84.0)	117,226	(10.8)	32,805	(3.0)	22,343	(2.1)	1,080,665
**Sum**	4,058,505	(79.9)	621,791	(12.3)	258,080	(5.1)	139,037	(2.8)	5,077,413

Percentages are relative to the total numbers of each cancer sites, and they may not add up to 1 because of rounding to 1 digit.

Supplementary Table S2. Characteristics of Female Breast Cancer by Race/ethnicity.

Characteristics	NH White N=1,322,385, %	NH Black N=193,481, %	Hispanic N=94,360, %	Asian N=54,271, %	*P*
**Follow up (months, median [IQR])**	60.8 [34.8, 93.0]	53.4 [29.9, 84.6]	55.4 [31.2, 86.0]	56.3 [32.6, 87.5]	<0.001
**Age (years)**					<0.001
18-44	11.1	16.3	21.4	20.6	
45-54	23.7	26.8	29.1	30.2	
55-64	26.5	27.0	24.3	24.7	
65-74	21.9	18.2	15.9	16.1	
>75	16.8	11.6	9.3	8.4	
**Year of Diagnosis**					<0.001
2004~2009	50.5	47.0	46.5	43.4	
2010~2014	49.5	53.0	53.5	56.6	
**Region**					<0.001
Northeast	21.8	16.5	18.0	20.0	
Midwest	24.6	18.3	7.6	9.9	
South	33.3	52.8	36.2	16.2	
West	16.1	5.1	28.3	45.1	
Unknown	4.3	7.4	9.9	8.7	
**Residence** [a]					<0.001
Metro	81.4	90.0	93.6	94.7	
Urban	13.7	6.9	3.9	2.1	
Rural	1.8	0.8	0.2	0.1	
Unknown	3.2	2.3	2.3	3.1	
**Household Income** [b]					<0.001
<$30000	8.6	31.2	21.0	4.6	
$30000-$34999	16.4	20.0	18.8	9.0	
$35000-$45999	26.5	23.0	26.8	20.8	
>$46000	46.0	23.4	31.3	63.3	
Unknown	2.5	2.4	2.0	2.4	
**Education (No High School Graduation)** [b]					<0.001
≥29%	9.8	31.4	41.6	14.2	
20%-28.9%	19.8	30.9	21.7	17.6	
14%-19.9%	27.0	19.1	16.4	22.0	
<14%	40.8	16.2	18.2	43.8	
Unknown	2.5	2.4	2.0	2.4	
**Socioeconomic Status**					<0.001
Low	17.4	46.4	37.2	10.5	
Intermediate	49.4	40.9	47.2	49.4	
High	33.2	12.7	15.5	40.0	
**Facility Type**					<0.001
Community	10.3	7.6	8.2	9.3	
Comprehensive Community	48.5	35.1	37.2	39.3	
Academic/Research	26.7	36.7	32.1	35.3	
Integrated Network	10.2	13.2	12.6	7.5	
Unknown	4.3	7.4	9.9	8.7	
**Insurance**					<0.001

Characteristics	NH White N=1,322,385, %	NH Black N=193,481, %	Hispanic N=94,360, %	Asian N=54,271, %	*P*
No Insurance	1.4	4.1	9.0	3.4	
Private Insurance	56.5	49.8	46.5	64.3	
Medicaid	3.9	12.7	18.2	10.7	
Medicare	35.5	29.9	21.5	18.8	
Other Government	0.9	1.3	0.9	1.2	
Unknown	1.7	2.3	3.8	1.7	
**Histology Grade**					<0.001
I	10.5	7.3	8.9	9.1	
II	17.0	15.0	17.0	18.7	
III	11.1	17.2	14.2	14.1	
Unknown	61.4	60.5	59.8	58.0	
**Histology type**					<0.001
Ductal	66.8	68.8	67.8	67.1	
Lobular	9.6	6.8	7.7	5.5	
Other	23.7	24.4	24.5	27.4	
**ER status**					<0.001
Positive	76.5	65.6	70.9	75.1	
Negative	16.2	27.1	19.8	17.6	
Unknown	7.2	7.3	9.3	7.3	
**PR status**					<0.001
Positive	66.1	55.1	61.1	65.3	
Negative	25.3	36.5	28.3	25.9	
Unknown	8.6	8.4	10.6	8.8	
**HER2 status**					<0.001
Positive	5.8	7.0	7.6	8.5	
Negative	32.5	33.0	32.6	32.8	
Unknown	61.7	60.0	59.8	58.7	
**Lymph Vascular Invasion**					<0.001
No	32.4	32.2	31.5	36.4	
Yes	6.6	7.3	8.1	7.5	
Unknown	61.1	60.5	60.3	56.1	
**TNM Stage**					<0.001
0	19.1	20.2	19.1	23.8	
I	40.9	30.1	32.0	36.1	
II	24.7	28.5	29.0	25.9	
III	8.3	11.8	11.7	8.1	
IV	3.5	5.6	3.9	2.6	
Unknown	3.5	3.7	4.4	3.5	
**Comorbidity (Charlson)**					<0.001
No	86.5	79.9	85.4	89.8	
Yes	13.5	20.1	14.6	10.2	
**Surgery**					<0.001
Breast Conserving	58.5	53.4	51.5	52.7	
Mastectomy	35.4	36.2	39.8	41.2	
No or Unknown	6.1	10.4	8.7	6.1	
**Radiation**					<0.001
No	42.3	43.8	46.0	45.1	
Yes	56.7	54.9	52.4	53.7	
Unknown	1.1	1.2	1.7	1.2	
**Chemotherapy**					<0.001
No	62.7	52.0	51.6	58.3	
Yes	34.3	44.6	44.5	38.1	
Unknown	3.1	3.4	3.9	3.6	
**Endocrine Therapy**					<0.001
No	38.1	47.3	42.9	39.6	
Yes	57.6	47.4	51.3	55.0	
Unknown	4.3	5.3	5.8	5.4	
**Immunotherapy**					<0.001
No	96.3	95.4	95.4	95.3	
Yes	2.2	2.6	3.0	3.1	
Unknown	1.5	2.0	1.6	1.6	
**Time-to-Treatment (days, median [IQR])**	23.0 [12.0, 37.0]	27.0 [11.0, 45.0]	28.0 [13.0, 8.0]	24.0 [13.0, 1.0]	<0.001

Abbreviation: IQR, Interquartile Range; ER, Estrogen Receptor; PR, Progesterone Receptor; HER2, Human Epidermal Growth Factor Receptor 2.

a. Residence was estimated by matching patients' Federal Information Processing Standards codes against US Department of Agriculture Economic Research Service records from 2003 or 2013, depending on the time of diagnosis.
b. Median annual household income and educational attainment were estimated by matching patients' zip codes against American Community Survey data from 2000 or 2012, depending on the time of diagnosis.
SI conversion factors: To convert miles to kilometers, multiply by 1.6.

Supplementary Table S3. Characteristics of Ovarian Cancer by Race/ethnicity.

Characteristics	NH White N=108,578, %	NH Black N=11,155, %	Hispanic N=8,474, %	Asian N=4,204, %	*P*
**Follow up (months, median [IQR])**	34.0 [14.6, 63.2]	26.6 [8.3, 53.6]	34.4 [15.1, 63.2]	37.4 [18.5, 65.8]	<0.001
**Age (years)**					<0.001
18-44	10.4	15.6	24.3	21.8	
45-54	19.1	20.8	23.3	28.4	
55-64	25.8	25.6	22.5	23.6	
65-74	22.8	20.9	16.7	15.7	
>75	21.9	17.1	13.2	10.6	
**Year of Diagnosis**					<0.001
2004~2009	53.1	50.5	49.0	44.0	
2010~2014	46.9	49.5	51.0	56.0	
**Region**					<0.001
Northeast	20.5	15.9	13.1	18.1	
Midwest	23.9	16.5	6.6	8.9	
South	33.7	52.2	34.4	17.4	
West	16.0	5.4	30.1	41.9	
Unknown	5.9	10.0	15.9	13.6	
**Residence**					<0.001
Metro	78.3	87.5	91.6	93.5	
Urban	15.7	8.9	5.3	2.7	
Rural	2.0	1.1	0.4	0.2	
Unknown	4.0	2.5	2.7	3.7	
**Household Income**					<0.001
<$30000	10.1	34.3	22.8	6.8	
$30000-$34999	18.0	21.3	19.8	10.2	
$35000-$45999	27.3	21.7	26.8	22.2	
>$46000	41.4	19.9	28.3	58.5	
Unknown	3.2	2.8	2.2	2.4	
**Education (No High School Graduation)**					<0.001
≥29%	11.3	34.7	45.6	17.7	
20%-28.9%	21.5	32.3	21.5	18.5	
14%-19.9%	27.2	17.0	15.0	22.6	
<14%	36.7	13.3	15.7	38.8	
Unknown	3.2	2.8	2.2	2.4	
**Socioeconomic Status**					<0.001
Low	20.0	51.0	40.3	13.9	
Intermediate	51.1	38.6	46.3	50.3	
High	28.9	10.4	13.4	35.8	
**Facility Type**					<0.001
Community	6.1	5.6	5.3	6.8	
Comprehensive Community	40.6	30.3	31.6	32.4	
Academic/Research	36.9	42.3	36.5	40.8	
Integrated Network	10.5	11.9	10.8	6.4	
Unknown	5.9	10.0	15.9	13.6	
**Insurance**					<0.001
No Insurance	3.5	7.2	15.3	7.6	
Private Insurance	47.8	39.0	38.6	57.5	
Medicaid	4.5	13.1	17.0	11.0	
Medicare	41.6	36.6	25.1	20.0	
Other Government	0.9	1.1	0.8	1.6	
Unknown	1.8	2.9	3.3	2.3	
**Histology Grade**					<0.001

Characteristics	NH White N=108,578, %	NH Black N=11,155, %	Hispanic N=8,474, %	Asian N=4,204, %	*P*
I	7.7	6.9	8.4	9.1	
II	12.4	10.4	12.2	12.5	
III	38.1	34.4	34.1	36.5	
IV	10.0	6.7	8.8	11.2	
Unknown	31.9	41.5	36.5	30.6	
**Histology type**					<0.001
Epithelial	88.0	77.9	81.3	86.3	
Other	12.0	22.1	18.7	13.7	
**Lymph Vascular Invasion**					<0.001
No	17.0	17.3	17.8	23.5	
Yes	7.6	6.4	8.1	8.3	
Unknown	75.5	76.2	74.2	68.2	
**TNM Stage**					<0.001
I	21.7	20.5	25.3	31.8	
II	8.5	7.2	8.1	9.5	
III	38.9	33.8	34.0	32.5	
IV	20.2	25.9	21.5	16.1	
Unknown	10.7	12.6	11.1	10.2	
**CA125**					<0.001
Negative	8.5	9.1	9.3	10.1	
Positive	67.4	61.0	61.2	64.6	
Unknown	24.1	29.9	29.5	25.4	
**Comorbidity (Charlson)**					<0.001
No	80.5	73.6	80.9	86.2	
Yes	19.5	26.4	19.1	13.8	
**Surgery**					<0.001
No	17.7	26.3	17.9	12.5	
Yes	82.1	73.5	81.9	87.4	
Unknown	0.2	0.2	0.2	0.2	
**Radiation**					<0.001
No	97.8	97.4	97.3	97.2	
Yes	1.0	1.3	1.1	1.3	
Unknown	1.2	1.3	1.6	1.5	
**Chemotherapy**					<0.001
No	27.6	34.0	31.3	29.3	
Yes	69.2	62.4	64.5	66.8	
Unknown	3.2	3.6	4.2	3.9	
**Time-to-Treatment (days, median [IQR])**	0.0 [0.0, 12.0]	0.0 [0.0, 13.0]	0.0 [0.0, 12.0]	0.0 [0.0, 10.0]	<0.001

Supplementary Table S4. Characteristics of Prostate Cancer by Race/ethnicity.

Characteristics	NH White N=826,152, %	NH Black N=152,571, %	Hispanic N=47,796, %	Asian N=18,563, %	*P*
**Follow up (months, median [IQR])**	65.7 [38.0, 96.9]	59.5 [32.8, 89.6]	59.3 [31.5, 89.9]	63.1 [36.1, 94.5]	<0.001
**Age (years)**					<0.001
18-44	0.6	1.4	1.1	0.4	
45-54	10.8	16.9	12.6	7.7	
55-64	36.0	40.5	35.1	30.8	
65-74	37.5	31.5	36.9	42.1	
>75	15.1	9.7	14.3	19.0	
**Year of Diagnosis**					<0.001
2004~2009	57.5	53.1	54.6	54.4	
2010~2014	42.5	46.9	45.4	45.6	
**Region**					<0.001
Northeast	21.3	18.5	23.0	20.5	
Midwest	27.3	19.8	10.1	10.7	
South	33.9	55.7	36.1	15.6	
West	17.4	5.9	30.7	53.2	
Unknown	0.1	0.1	0.1	0.0	
**Residence**					<0.001
Metro	77.9	87.9	92.4	94.3	
Urban	16.7	8.7	4.9	2.7	

Characteristics	NH White N=826,152, %	NH Black N=152,571, %	Hispanic N=47,796, %	Asian N=18,563, %	*P*
Rural	2.4	1.1	0.3	0.1	
Unknown	3.0	2.3	2.4	2.8	
**Household Income**					<0.001
<$30000	8.9	30.8	23.3	5.0	
$30000-$34999	16.9	19.6	18.3	8.3	
$35000-$45999	26.8	23.1	26.0	20.6	
>$46000	44.5	23.9	30.0	63.6	
Unknown	2.9	2.6	2.3	2.4	
**Education (No High School Graduation)**					<0.001
≥29%	9.9	31.9	43.3	14.5	
20%-28.9%	19.9	30.7	21.4	18.5	
14%-19.9%	26.6	18.4	15.3	21.1	
<14%	40.8	16.4	17.7	43.5	
Unknown	2.8	2.6	2.3	2.4	
**Socioeconomic Status**					<0.001
Low	17.7	46.0	39.2	10.4	
Intermediate	49.6	40.9	45.7	49.4	
High	32.7	13.1	15.1	40.1	
**Facility Type**					<0.001
Community	8.6	8.5	8.2	8.6	
Comprehensive Community	45.4	36.3	38.5	35.6	
Academic/Research	35.9	42.1	40.5	49.2	
Integrated Network	10.0	13.0	12.7	6.6	
Unknown	0.1	0.1	0.1	0.0	
**Insurance**					<0.001
No Insurance	1.1	4.1	6.6	2.9	
Private Insurance	48.6	46.9	41.4	46.8	
Medicaid	1.2	6.1	9.1	6.7	
Medicare	45.4	37.5	38.4	41.2	
Government Insurance	1.6	2.9	1.2	0.6	
Unknown	2.2	2.4	3.3	1.7	
**Gleason Grade**					<0.001
1	39.6	35.7	37.4	34.0	
2	29.5	29.8	26.7	27.8	
3	11.1	11.4	10.8	13.4	
4	6.7	7.6	7.8	9.4	
5	6.5	6.5	7.7	8.9	
Unknown	6.7	9.0	9.6	6.4	
**Histology type**					<0.001
Adenocarcinoma	97.3	97.1	97.1	97.0	
Other	2.7	2.9	2.9	3.0	
**Lymph Vascular Invasion**					<0.001
No	23.4	23.3	22.4	23.2	
Yes	1.5	1.3	1.6	1.4	
Unknown	75.1	75.4	76.0	75.3	
**TNM Stage**					<0.001
1	10.2	10.1	10.3	9.4	
2	70.3	70.1	67.6	69.6	
3	10.2	8.3	10.0	11.3	
4	3.5	5.5	5.8	4.5	
Unknown	5.8	5.9	6.3	5.2	
**PSA**					<0.001
Negative	8.0	4.1	5.8	5.6	
Positive	83.0	88.3	84.2	87.8	
Unknown	8.9	7.7	10.0	6.6	
**Comorbidity (Charlson)**					<0.001
No	85.3	80.4	83.0	84.1	
Yes	14.7	19.6	17.0	15.9	
**Surgery**					<0.001
No	44.1	54.0	48.8	50.3	
Yes	55.7	45.7	50.7	49.5	

Characteristics	NH White N=826,152, %	NH Black N=152,571, %	Hispanic N=47,796, %	Asian N=18,563, %	*P*
Unknown	0.2	0.3	0.5	0.2	
**Radiation**					<0.001
No	63.2	58.1	62.5	59.2	
Yes	35.9	40.8	36.0	39.8	
Unknown	0.9	1.1	1.5	1.0	
**Hormone Therapy**					<0.001
No	76.9	71.6	72.1	70.4	
Yes	19.7	24.0	23.4	25.6	
Unknown	3.4	4.4	4.5	4.1	
**Chemotherapy**					<0.001
No	96.7	95.7	95.1	95.8	
Yes	0.6	0.6	0.7	0.6	
Unknown	2.7	3.7	4.2	3.7	
**Time-to-Treatment (days, median [IQR])**	57.0 [26.0, 85.0]	60.8 [23.0, 95.0]	58.0 [21.0, 87.0]	60.8 [26.0, 85.0]	<0.001

Abbreviation: PSA, Prostate Specific Antigen..

Supplementary Table S5. Characteristics of Colorectal Cancer by Race/ethnicity.

Characteristics	NH White N=635,569, %	NH Black N=100,124, %	Hispanic N=46,270, %	Asian N=24,413, %	*P*
**Follow up (months, median [IQR])**	40.7 [17.5, 75.2]	36.2 [15.2, 69.1]	38.8 [17.5, 70.8]	41.2 [20.2, 73.3]	<0.001
**Age (years)**					<0.001
18-44	5.8	7.8	11.8	9.0	
45-54	14.7	20.8	20.1	18.4	
55-64	21.8	27.7	25.3	25.0	
65-74	24.7	23.3	22.3	24.2	
>75	32.9	20.4	20.5	23.5	
**SEX**					<0.001
Male	51.5	46.6	54.2	51.6	
Female	48.5	53.4	45.8	48.4	
**Year of Diagnosis**					<0.001
2004~2009	54.1	51.6	49.3	47.7	
2010~2014	45.9	48.4	50.7	52.3	
**Region**					<0.001
Northeast	22.0	16.1	17.9	19.8	
Midwest	26.6	20.1	7.9	10.6	
South	34.5	55.0	39.6	16.6	
West	14.0	4.9	27.8	48.4	
Unknown	2.8	3.8	6.7	4.6	
**Residence**					<0.001
Metro	78.6	88.2	92.6	94.5	
Urban	15.7	8.5	4.8	2.2	
Rural	2.2	1.0	0.4	0.2	
Unknown	3.4	2.4	2.2	3.1	
**Household Income**					<0.001
<$30000	11.1	36.0	24.8	6.4	
$30000-$34999	19.0	20.6	20.7	11.2	
$35000-$45999	27.7	21.7	26.0	23.6	
>$46000	39.4	19.3	26.5	56.4	
Unknown	2.8	2.5	2.1	2.4	
**Education (No High School Graduation)**					<0.001
≥29%	12.7	36.3	46.2	18.6	
20%-28.9%	23.0	31.3	21.9	19.6	
14%-19.9%	27.5	16.8	15.0	21.2	
<14%	34.1	13.2	14.8	38.2	
Unknown	2.8	2.5	2.1	2.4	
**Socioeconomic Sta- tus**					<0.001
Low	22.0	52.0	42.8	14.3	
Intermediate	51.1	38.0	44.8	51.1	
High	26.9	10.1	12.4	34.6	
**Facility Type**					<0.001

Characteristics	NH White N=635,569, %	NH Black N=100,124, %	Hispanic N=46,270, %	Asian N=24,413, %	*P*
Community	13.1	9.9	9.2	12.3	
Comprehensive Community	48.2	36.0	40.0	38.9	
Academic/Research	25.5	37.5	31.3	36.6	
Integrated Network	10.3	12.9	12.8	7.6	
Unknown	2.8	3.8	6.7	4.6	
**Insurance**					<0.001
No Insurance	2.7	7.0	10.8	5.7	
Private Insurance	38.3	36.5	36.4	44.4	
Medicaid	3.6	10.7	13.6	11.0	
Medicare	52.8	42.5	35.6	36.4	
Other Government	0.9	1.2	0.7	0.8	
Unknown	1.7	2.2	2.8	1.7	
**Primary Site**					<0.001
Colon	68.9	74.2	65.2	63.0	
Rectum	23.1	19.6	25.7	26.5	
Rect0sigmoid	8.0	6.2	9.1	10.4	
**Histology Grade**					<0.001
I	9.7	10.4	10.1	9.1	
II	56.6	55.1	55.9	58.2	
III	15.1	12.1	14.3	13.8	
IV	1.9	1.2	1.6	1.5	
Unknown	16.6	21.2	18.2	17.4	
**Histology type**					<0.001
Adenocarcinoma	93.3	90.3	91.8	92.0	
Other	6.7	9.7	8.2	8.0	
**Lymph Vascular Invasion**					<0.001
No	24.8	24.9	25.7	27.3	
Yes	9.1	8.9	9.5	10.3	
Unknown	66.1	66.2	64.8	62.4	
**TNM Stage**					<0.001
0	5.9	6.9	5.4	5.1	
I	21.5	18.8	18.6	20.3	
II	23.4	19.7	22.2	21.4	
III	23.7	22.9	25.9	27.2	
IV	17.4	21.9	19.0	16.8	
Unknown	8.1	9.8	9.0	9.1	
**CEA**					<0.001
Positive (elevated)	23.5	29.6	25.6	26.9	
Negative (normal)	28.1	21.2	24.7	25.0	
Unknown	48.5	49.2	49.7	48.2	
**Circumferential Resection Margin**					<0.001
Positive (<1mm)	3.8	3.6	4.5	4.6	
Negative (≥1mm)	26.5	25.2	27.0	29.1	
Unknown	69.6	71.2	68.5	66.3	
**Comorbidity (Charlson)**					<0.001
No	72.6	70.5	74.5	79.8	
Yes	27.4	29.5	25.5	20.2	
**Surgery**					<0.001
No	13.8	18.4	16.8	14.1	
Yes	86.0	81.4	82.9	85.7	
Unknown	0.2	0.2	0.3	0.1	
**Radiation**					<0.001
No	83.3	86.9	80.9	81.5	
Yes	15.6	11.9	17.5	17.0	
Unknown	1.0	1.2	1.5	1.5	
**Chemotherapy**					<0.001
No	56.2	55.4	48.9	51.1	
Yes	40.4	40.3	46.1	44.4	
Unknown	3.4	4.3	4.9	4.6	
**Time-to-Treatment (days, median [IQR])**	8.0 [0.0, 25.0]	5.0 [0.0, 26.0]	9.0 [0.0, 28.0]	11.0 [0.0, 26.0]	<0.001

Abbreviation: CEA, Carcinoembryonic Antigen.

Supplementary Table S6. Characteristics of Pancreatic Cancer by Race/ethnicity.

Characteristics	NH White N=182,538, %	NH Black N=28,935, %	Hispanic N=12,920, %	Asian N=5,970, %	*P*
**Follow up (months, median [IQR])**	6.7 [2.1, 16.6]	6.0 [1.9, 15.4]	6.6 [2.1, 17.5]	7.2 [2.3, 18.8]	<0.001
**Age (years)**					<0.001
18-44	2.9	4.6	6.6	4.8	
45-54	10.6	15.9	14.7	10.5	
55-64	24.3	28.6	24.4	22.9	
65-74	29.0	26.7	28.0	28.5	
>75	33.3	24.3	26.3	33.2	
**SEX**					
Male	51.4	44.4	50.1	48.1	
Female	48.6	55.6	49.9	51.9	
**Year of Diagnosis**					<0.001
2004~2009	47.5	45.6	45.7	42.4	
2010~2014	52.5	54.4	54.3	57.6	
**Region**					<0.001
Northeast	22.6	17.0	17.3	19.5	
Midwest	25.7	19.8	8.2	10.1	
South	35.2	55.7	39.4	16.4	
West	15.3	5.5	31.6	51.8	
Unknown	1.2	2.0	3.5	2.3	
**Residence**					<0.001
Metro	77.1	86.5	91.1	93.0	
Urban	16.3	9.1	5.4	2.6	
Rural	2.2	1.2	0.4	0.2	
Unknown	4.4	3.3	3.0	4.2	
**Household Income**					<0.001
<$30000	11.0	36.8	23.6	6.4	
$30000-$34999	19.2	20.8	20.3	11.1	
$35000-$45999	27.4	20.8	26.3	23.2	
>$46000	38.9	18.6	26.9	56.1	
Unknown	3.5	3.0	2.9	3.3	
**Education (No High School Graduation)**					<0.001
≥29%	12.0	36.3	45.8	17.8	
20%-28.9%	22.4	31.1	21.0	20.0	
14%-19.9%	28.0	17.1	14.9	22.0	
<14%	34.1	12.6	15.4	36.9	
Unknown	3.5	3.0	2.9	3.3	
**Socioeconomic Status**					<0.001
Low	21.6	52.6	41.2	14.0	
Intermediate	51.7	37.8	45.8	52.8	
High	26.7	9.6	12.9	33.1	
**Facility Type**					<0.001
Community	7.8	6.7	6.3	9.3	
Comprehensive Community	39.0	30.0	35.3	34.2	
Academic/Research	41.7	47.8	41.8	47.5	
Integrated Network	10.3	13.6	13.1	6.8	
Unknown	1.2	2.0	3.5	2.3	
**Insurance**					<0.001
No Insurance	2.6	5.8	10.0	4.9	
Private Insurance	33.9	30.3	29.3	35.1	
Medicaid	3.8	11.4	14.5	10.4	
Medicare	56.3	48.8	41.9	46.9	
Other Government	1.1	1.3	0.6	0.9	
Unknown	2.3	2.3	3.6	1.8	
**Histology Grade**					<0.001
I	6.3	6.3	6.5	7.1	
II	15.0	14.3	14.8	15.4	
III	15.1	14.1	15.4	14.0	
IV	0.9	0.7	0.9	1.1	
Unknown	62.6	64.6	62.4	62.4	
**Histology type**					<0.001
Epithelial	75.8	75.7	73.4	73.3	

Characteristics	NH White N=182,538, %	NH Black N=28,935, %	Hispanic N=12,920, %	Asian N=5,970, %	*P*
Other	24.2	24.3	26.6	26.7	
**Lymph Vascular Invasion**					<0.001
No	9.5	10.2	9.0	9.7	
Yes	5.6	4.6	5.0	4.9	
Unknown	85.0	85.2	86.0	85.3	
**TNM Stage**					<0.001
0	0.7	0.5	0.5	0.9	
I	9.1	8.7	8.6	9.8	
II	24.9	22.1	23.6	23.5	
III	9.9	11.1	9.8	12.0	
IV	43.5	45.9	44.7	42.7	
Unknown	11.8	11.8	12.8	11.2	
**CA 19-9**					<0.001
≤37.0 U/ml	8.4	13.0	11.1	10.9	
>37.0 U/ml	25.0	21.9	23.2	25.3	
Unknown	66.6	65.1	65.7	63.9	
**Comorbidity (Charlson)**					<0.001
No	67.8	61.8	64.8	71.3	
Yes	32.2	38.2	35.2	28.7	
**Surgery**					<0.001
No	75.9	80.1	77.3	76.5	
Yes	23.8	19.5	22.2	23.1	
Unknown	0.4	0.5	0.5	0.4	
**Radiation**					<0.001
No	81.6	82.3	84.7	83.0	
Yes	17.7	16.8	14.2	16.0	
Unknown	0.7	0.9	1.1	0.9	
**Chemotherapy**					<0.001
No	46.2	49.2	49.7	48.4	
Yes	50.2	46.9	45.2	47.2	
Unknown	3.7	4.0	5.1	4.4	
**Time-to-Treatment (days, median [IQR])**	12.0 [0.0, 26.0]	11.0 [0.0, 29.0]	9.0 [0.0, 27.0]	9.0 [0.0, 25.1]	<0.001

Abbreviation: CA 19-9, Carbohydrate Antigen 19-9.

Supplementary Table S7. Characteristics of Liver Cancer by Race/ethnicity.

Characteristics	NH White N=74,992, %	NH Black N=18,299, %	Hispanic N=15,455, %	Asian N=9,273, %	*P*
**Follow up (months, median [IQR])**	10.4 [2.6, 29.8]	8.7 [2.2, 26.2]	11.6 [2.6, 31.2]	14.9 [3.1, 40.6]	<0.001
**Age (years)**					<0.001
18-44	2.7	5.0	4.5	7.5	
45-54	18.5	22.2	22.6	17.8	
55-64	36.2	46.2	35.1	29.8	
65-74	23.4	18.4	22.8	26.1	
>75	19.2	8.2	14.9	18.8	
**SEX**					<0.001
Male	75.1	73.7	73.6	71.6	
Female	24.9	26.3	26.4	28.4	
**Year of Diagnosis**					<0.001
2004~2009	45.5	43.7	43.8	48.2	
2010~2014	54.5	56.3	56.2	51.8	
**Region**					<0.001
Northeast	20.7	19.1	18.7	20.9	
Midwest	22.3	20.1	7.8	10.0	
South	39.0	50.6	38.9	19.3	
West	16.6	7.4	32.7	46.3	
Unknown	1.3	2.9	2.0	3.5	
**Residence**					<0.001
Metro	78.4	90.5	90.9	93.2	
Urban	15.7	6.3	6.2	2.0	
Rural	1.8	0.6	0.4	0.1	
Unknown	4.0	2.7	2.5	4.7	
**Household Income**					<0.001
<$30000	13.8	40.8	30.7	9.9	
$30000-$34999	21.1	20.8	22.4	13.3	
$35000-$45999	28.1	19.9	23.8	25.7	
>$46000	33.8	16.0	20.4	47.5	
Unknown	3.3	2.5	2.7	3.6	
**Education (No High School Graduation)**					<0.001
≥29%	15.5	38.5	52.1	24.3	
20%-28.9%	25.7	31.5	20.9	21.3	
14%-19.9%	27.3	16.0	13.0	20.7	
<14%	28.3	11.5	11.3	30.0	
Unknown	3.2	2.5	2.7	3.6	
**Socioeconomic Status**					<0.001
Low	26.4	56.0	50.1	19.9	
Intermediate	51.9	35.7	40.8	53.7	
High	21.7	8.3	9.1	26.4	
**Facility Type**					<0.001
Community	6.0	5.4	5.0	4.6	
Comprehensive Community	29.8	21.6	27.6	28.0	
Academic/Research	52.1	57.9	56.0	57.0	
Integrated Network	10.8	12.3	9.4	7.0	
Unknown	1.3	2.9	2.0	3.5	
**Insurance**					<0.001
No Insurance	4.7	9.2	10.8	7.2	
Private Insurance	35.0	28.8	26.6	37.2	
Medicaid	10.5	22.8	20.4	16.7	
Medicare	45.8	34.5	38.0	36.1	
Other Government	1.8	2.3	1.1	0.7	
Unknown	2.2	2.5	3.1	2.1	
**Histology Grade**					<0.001
I	13.1	11.6	11.4	10.1	
II	17.9	15.9	13.7	18.5	
III	9.7	9.7	7.3	11.4	
IV	1.0	0.8	1.1	1.0	
Unknown	58.2	62.0	66.4	59.0	
**Histology type**					<0.001

Characteristics	NH White N=74,992, %	NH Black N=18,299, %	Hispanic N=15,455, %	Asian N=9,273, %	*P*
Hepatocelluar Carcinoma	87.4	91.4	92.2	92.4	
Other	12.6	8.6	7.8	7.6	
**Lymph Vascular Invasion**					<0.001
No	10.5	11.0	9.1	10.2	
Yes	2.6	2.3	1.7	3.8	
Unknown	86.8	86.7	89.2	86.0	
**TNM Stage**					<0.001
I	28.2	26.0	28.6	29.3	
II	18.3	16.2	18.9	18.0	
III	19.5	22.4	18.1	21.9	
IV	12.0	14.1	12.1	11.3	
Unknown	22.1	21.3	22.2	19.5	
**AFP**					<0.001
Positive	50.1	62.6	55.5	58.3	
Negative	21.3	12.1	18.7	18.0	
Unknown	28.6	25.2	25.8	23.7	
**Fibrosis Score**					<0.001
F0 (0-4)	4.4	4.4	3.5	7.9	
F1 (5-6)	14.2	12.8	15.9	14.6	
unknown	81.4	82.7	80.7	77.5	
**INR**					<0.001
≤1.3	22.1	21.8	21.0	23.3	
>1.3	10.9	10.9	11.8	6.6	
unknown	67.0	67.3	67.1	70.1	
**Comorbidity (Charlson)**					<0.001
No	47.7	49.1	44.0	59.6	
Yes	52.3	50.9	56.0	40.4	
**Surgery**					<0.001
No	71.1	77.5	75.8	65.1	
Yes	28.5	22.0	23.6	34.6	
Unknown	0.4	0.5	0.6	0.3	
**Radiation**					<0.001
No	90.0	91.5	93.0	92.1	
Yes	9.3	7.6	6.0	6.6	
Unknown	0.7	0.9	0.9	1.3	
**Chemotherapy**					<0.001
No	59.1	59.0	56.4	57.0	
Yes	38.1	37.9	40.4	39.4	
Unknown	2.8	3.1	3.2	3.6	
**Time-to-Treatment (days, median [IQR])**	21.0 [0.0, 49.6]	17.0 [0.0, 49.6]	17.9 [0.0, 50.0]	21.0 [0.0, 48.0]	<0.001

Abbreviation: AFP, Alpha Fetoprotein; INR, International Normalized Ratio.

Supplementary Table S8. Characteristics of Lung Cancer by Race/ethnicity.

Characteristics	NH White N=908,291 %	NH Black N=117,226, %	Hispanic N=32,805, %	Asian N=22,343, %	*P*
**Follow up (months, median [IQR])**	10.7 [3.1, 29.7]	10.2 [3.2, 27.0]	10.6 [3.0, 29.3]	14.7 [4.1, 35.5]	<0.001
**Age (years)**					<0.001
18-44	1.9	2.8	4.5	4.0	
45-54	10.1	16.5	11.9	11.3	
55-64	23.8	30.5	24.3	22.5	
65-74	33.6	29.5	31.2	30.4	
>75	30.6	20.7	28.1	31.8	
**SEX**					<0.001
Male	51.7	53.5	56.9	56.6	
Female	48.3	46.5	43.1	43.4	
**Year of Diagnosis**					<0.001
2004~2009	53.7	51.7	51.3	47.3	
2010~2014	46.3	48.3	48.7	52.7	
**Region**					<0.001
Northeast	20.6	15.6	19.5	21.1	
Midwest	26.4	22.6	8.0	9.9	
South	39.4	55.7	42.9	17.0	
West	13.0	5.3	27.2	50.1	
Unknown	0.6	0.9	2.3	1.8	
**Residence**					<0.001
Metro	75.3	87.0	90.7	93.2	
Urban	18.2	9.3	5.8	2.6	
Rural	2.5	1.2	0.4	0.2	
Unknown	4.0	2.5	3.1	4.0	
**Household Income**					<0.001
<$30000	13.4	41.1	26.2	7.7	
$30000-$34999	21.1	21.5	20.9	12.6	
$35000-$45999	28.6	20.0	25.5	24.8	
>$46000	33.6	14.9	24.6	51.8	
Unknown	3.2	2.5	2.8	3.1	
**Education (No High School Graduation)**					<0.001
≥29%	14.9	40.2	45.8	21.6	
20%-28.9%	25.4	31.5	22.2	20.6	
14%-19.9%	27.7	15.4	15.1	21.7	
<14%	28.9	10.5	14.1	33.0	
Unknown	3.2	2.5	2.8	3.1	
**Socioeconomic Status**					<0.001
Low	26.0	57.5	44.4	16.7	
Intermediate	51.9	35.0	44.0	53.7	
High	22.1	7.5	11.6	29.6	
**Facility Type**					<0.001
Community	12.1	8.9	8.7	10.0	
Comprehensive Community	48.8	35.3	40.7	37.6	
Academic/Research	28.0	42.0	33.8	42.3	
Integrated Network	10.5	12.9	14.6	8.3	
Unknown	0.6	0.9	2.3	1.8	
**Insurance**					<0.001
No Insurance	3.1	6.7	8.6	5.0	
Private Insurance	28.2	25.3	24.9	32.3	
Medicaid	5.2	14.0	14.2	12.4	
Medicare	59.9	49.8	48.3	47.5	
Other Government	1.4	1.6	0.8	1.0	
Unknown	2.1	2.7	3.2	1.8	
**Histology Grade**					<0.001
I	4.9	4.0	5.9	6.6	
II	15.7	14.4	14.6	17.3	
III	25.6	26.9	24.6	22.2	
IV	4.7	3.3	4.0	2.4	
Unknown	49.1	51.3	50.9	51.5	
**Histology type**					<0.001

Characteristics	NH White N=908,291 %	NH Black N=117,226, %	Hispanic N=32,805, %	Asian N=22,343, %	*P*
NSCLC	78.2	83.0	80.8	86.4	
SCLC	14.9	10.1	11.5	7.1	
Other	6.9	6.9	7.7	6.4	
**Lymph Vascular Invasion**					<0.001
No	11.1	10.5	10.5	11.5	
Yes	2.4	2.2	2.5	3.1	
Unknown	86.5	87.3	87.0	85.4	
**TNM Stage**					<0.001
0	0.2	0.2	0.2	0.2	
I	20.8	16.5	17.1	18.2	
II	7.4	6.7	6.5	6.4	
III	22.4	24.6	21.1	20.9	
IV	40.6	43.6	45.0	46.3	
Unknown	8.6	8.3	10.1	7.9	
**Separate Tumor Nodule**					<0.001
No	34.3	34.7	33.0	35.9	
Yes	8.1	9.2	10.0	11.7	
Unknown	57.6	56.1	57.0	52.4	
**Comorbidity (Charlson)**					<0.001
No	57.4	57.9	61.6	72.2	
Yes	42.6	42.1	38.4	27.8	
**Surgery**					<0.001
No	75.1	80.8	77.1	75.0	
Yes	24.6	18.8	22.3	24.6	
Unknown	0.3	0.4	0.6	0.4	
**Radiation**					<0.001
No	58.6	56.3	63.9	62.9	
Yes	40.6	42.9	34.8	35.6	
Unknown	0.8	0.8	1.3	1.5	
**Chemotherapy**					<0.001
No	50.8	49.8	51.2	47.1	
Yes	46.5	46.9	44.4	49.0	
Unknown	2.7	3.3	4.4	3.9	
**Time-to-Treatment (days, median [IQR])**	15.0 [0.0, 34.0]	16.0 [0.0, 38.0]	13.0 [0.0, 34.0]	17.0 [0.0, 34.8]	<0.001

Supplementary Table S9. Multivariable-adjusted HRs and 95% CIs for Total Mortality in Association with Race/ethnicity of Major Cancer Types According to Stage.

	HR (95% CI)						
	**Breast Cancer**	**Ovarian Cancer**	**Prostate Cancer**	**Colorectal Cancer**	**Pancreatic Cancer**	**Liver Cancer**	**Lung Cancer**
**Stage I**							
NH White	1.00 (reference)	1.00 (reference)	1.00 (reference)	1.00 (reference)	1.00 (reference)	1.00 (reference)	1.00 (reference)
NH Black	1.19 (1.16 to 1.22)	1.39 (1.25 to 1.54)	1.34 (1.26 to 1.43)	1.21 (1.18 to 1.25)	1.07 (1.01 to 1.13)	1.02 (0.98 to 1.07)	1.01 (0.99 to 1.03)
Hispanic	0.80 (0.76 to 0.84)	0.95 (0.83 to 1.09)	0.77 (0.68 to 0.88)	0.85 (0.80 to 0.89)	0.93 (0.86 to 1.02)	0.88 (0.84 to 0.92)	0.82 (0.79 to 0.86)
Asian	0.62 (0.58 to 0.66)	0.85 (0.71 to 1.02)	0.57 (0.45 to 0.71)	0.67 (0.63 to 0.73)	0.82 (0.73 to 0.92)	0.65 (0.61 to 0.69)	0.70 (0.66 to 0.74)
**Stage II**							
NH White	1.00 (reference)	1.00 (reference)	1.00 (reference)	1.00 (reference)	1.00 (reference)	1.00 (reference)	1.00 (reference)
NH Black	1.22 (1.19 to 1.25)	1.21 (1.08 to 1.37)	1.37 (1.35 to 1.40)	1.17 (1.14 to 1.20)	1.01 (0.98 to 1.04)	0.99 (0.94 to 1.04)	0.99 (0.96 to 1.02)
Hispanic	0.79 (0.76 to 0.82)	0.87 (0.75 to 1.02)	0.80 (0.77 to 0.83)	0.88 (0.85 to 0.92)	0.80 (0.77 to 0.84)	0.81 (0.77 to 0.85)	0.86 (0.81 to 0.91)
Asian	0.65 (0.61 to 0.69)	0.75 (0.60 to 0.95)	0.66 (0.62 to 0.69)	0.75 (0.70 to 0.79)	0.85 (0.80 to 0.90)	0.68 (0.63 to 0.73)	0.78 (0.72 to 0.84)
**Stage III**							
NH White	1.00 (reference)	1.00 (reference)	1.00 (reference)	1.00 (reference)	1.00 (reference)	1.00 (reference)	1.00 (reference)
NH Black	1.28 (1.25 to 1.31)	1.20 (1.15 to 1.25)	1.35 (1.28 to 1.43)	1.16 (1.14 to 1.19)	0.99 (0.95 to 1.03)	0.98 (0.94 to 1.01)	0.97 (0.95 to 0.98)
Hispanic	0.83 (0.80 to 0.86)	0.84 (0.79 to 0.88)	0.84 (0.76 to 0.92)	0.89 (0.86 to 0.92)	0.83 (0.78 to 0.88)	0.80 (0.76 to 0.84)	0.83 (0.81 to 0.86)
Asian	0.74 (0.69 to 0.78)	0.81 (0.75 to 0.88)	0.67 (0.58 to 0.78)	0.80 (0.76 to 0.83)	0.88 (0.82 to 0.96)	0.85 (0.80 to 0.89)	0.76 (0.74 to 0.79)
**Stage IV**							
NH White	1.00 (reference)	1.00 (reference)	1.00 (reference)	1.00 (reference)	1.00 (reference)	1.00 (reference)	1.00 (reference)
NH Black	1.18 (1.15 to 1.21)	1.14 (1.10 to 1.20)	1.05 (1.02 to 1.08)	1.05 (1.03 to 1.07)	1.03 (1.01 to 1.05)	0.95 (0.91 to 1.00)	0.95 (0.94 to 0.96)
Hispanic	0.85 (0.82 to 0.89)	0.84 (0.79 to 0.89)	0.82 (0.78 to 0.86)	0.83 (0.81 to 0.85)	0.86 (0.83 to 0.88)	0.78 (0.74 to 0.82)	0.77 (0.76 to 0.79)
Asian	0.86 (0.80 to 0.92)	0.83 (0.76 to 0.92)	0.74 (0.68 to 0.81)	0.86 (0.83 to 0.89)	0.89 (0.86 to 0.93)	0.90 (0.84 to 0.96)	0.69 (0.68 to 0.71)
***P* for Interaction**	$< 2.20\times10^{-16}$	$< 2.20\times10^{-16}$	4.39×10^{-11}	$< 2.20\times10^{-16}$	0.04	2.16×10^{-12}	$< 2.20\times10^{-16}$

Adjusted for age, sex (if applicable), biological (histology type, grade, LVI, comorbidity; ER, PR, HER2 for breast cancer; CA 125 for ovarian cancer; PSA and Gleason grade for prostate cancer; CA 19-9 for pancreatic cancer; AFP, Fibrosis Score, INR for liver cancer; separate tumor nodules for lung cancer; primary site,

CEA, circumferential resection margin for colorectal cancer), treatment factors (surgery, chemotherapy, interval between diagnosis to first treatment; endocrine therapy, radiation, immunotherapy, if applicable).
P values for interaction between racial/ethnic groups and TNM stages were derived from log likelihood test.
Abbreviation: HR, Hazard Ratio; CI, Confidence Interval.

Supplementary Table S10. Multivariable-adjusted HRs and 95% CIs for Total Mortality of Major Cancer Types According to SEX.

	Colorectal Cancer		Pancreatic Cancer		Liver Cancer		Lung Cancer	
	Male	Female	Male	Female	Male	Female	Male	Female
				Race/ethnicity				
NH White	1.00 (reference)	1.00 (reference)	1.00 (reference)	1.00 (reference)	1.00 (reference)	1.00 (reference)	1.00 (reference)	1.00 (reference)
NH Black	1.16 (1.15 to 1.18)	1.06 (1.04 to 1.07)	1.06 (1.04 to 1.08)	1.01 (0.99 to 1.03)	1.00 (0.98 to 1.02)	0.99 (0.95 to 1.03)	0.98 (0.97 to 0.99)	0.97 (0.96 to 0.98)
Hispanic	0.87 (0.86 to 0.89)	0.86 (0.84 to 0.88)	0.89 (0.86 to 0.91)	0.83 (0.81 to 0.86)	0.82 (0.80 to 0.84)	0.83 (0.79 to 0.86)	0.82 (0.80 to 0.83)	0.80 (0.78 to 0.82)
Asian	0.78 (0.76 to 0.81)	0.81 (0.78 to 0.83)	0.86 (0.83 to 0.90)	0.89 (0.85 to 0.92)	0.76 (0.73 to 0.78)	0.82 (0.78 to 0.87)	0.73 (0.72 to 0.75)	0.71 (0.69 to 0.73)

Adjusted for age, biological (histology type, grade, TNM stage, LVI, comorbidity; ER, PR, HER2 for breast cancer; CA 125 for ovarian cancer; PSA and Gleason grade for prostate cancer; CA 19-9 for pancreatic cancer; AFP, Fibrosis Score, INR for liver cancer; separate tumor nodules for lung cancer; primary site, CEA, circumferential resection margin for colorectal cancer), treatment factors (surgery, chemotherapy, interval between diagnosis to first treatment; endocrine therapy, radiation, immunotherapy, if applicable). Abbreviation: HR, Hazard Ratio; CI, Confidence Interval.

Supplementary Table S11. Multivariable-adjusted HRs and 95% CIs for 3-year Mortality Associated with Race/ethnicity According to Socioeconomic Status (SES) in Major Cancer Types.

	HR (95% CI)						
	**Breast Cancer**	**Ovarian Cancer**	**Prostate Cancer**	**Colorectal Cancer**	**Pancreatic Cancer**	**Liver Cancer**	**Lung Cancer**
**High SES**							
NH White	1.00 (reference)	1.00 (reference)	1.00 (reference)	1.00 (reference)	1.00 (reference)	1.00 (reference)	1.00 (reference)
NH Black	1.34 (1.28 to 1.40)	1.22 (1.11 to 1.34)	1.28 (1.19 to 1.37)	1.15 (1.10 to 1.19)	1.00 (0.95 to 1.04)	0.98 (0.91 to 1.04)	0.95 (0.93 to 0.98)
Hispanic	0.84 (0.78 to 0.91)	0.86 (0.77 to 0.96)	0.87 (0.77 to 0.98)	0.92 (0.87 to 0.97)	0.94 (0.89 to 1.00)	0.84 (0.79 to 0.91)	0.84 (0.80 to 0.87)
Asian	0.76 (0.70 to 0.82)	0.81 (0.72 to 0.90)	0.69 (0.62 to 0.78)	0.82 (0.78 to 0.86)	0.94 (0.89 to 0.99)	0.79 (0.74 to 0.83)	0.72 (0.70 to 0.74)
**Intermediate SES**							
NH White	1.00 (reference)	1.00 (reference)	1.00 (reference)	1.00 (reference)	1.00 (reference)	1.00 (reference)	1.00 (reference)
NH Black	1.17 (1.15 to 1.20)	1.10 (1.04 to 1.15)	1.23 (1.19 to 1.27)	1.04 (1.02 to 1.06)	1.01 (0.99 to 1.03)	0.95 (0.92 to 0.98)	0.95 (0.94 to 0.96)
Hispanic	0.77 (0.74 to 0.80)	0.82 (0.77 to 0.87)	0.81 (0.76 to 0.86)	0.81 (0.79 to 0.84)	0.85 (0.82 to 0.88)	0.84 (0.81 to 0.87)	0.82 (0.81 to 0.84)
Asian	0.73 (0.68 to 0.77)	0.81 (0.74 to 0.88)	0.71 (0.64 to 0.77)	0.78 (0.75 to 0.81)	0.87 (0.84 to 0.91)	0.81 (0.78 to 0.85)	0.70 (0.69 to 0.72)
**Low SES**							
NH White	1.00 (reference)	1.00 (reference)	1.00 (reference)	1.00 (reference)	1.00 (reference)	1.00 (reference)	1.00 (reference)
NH Black	1.14 (1.11 to 1.17)	1.16 (1.10 to 1.21)	1.16 (1.13 to 1.20)	1.01 (0.99 to 1.03)	0.97 (0.95 to 1.00)	0.98 (0.95 to 1.01)	0.94 (0.93 to 0.95)
Hispanic	0.70 (0.67 to 0.73)	0.76 (0.71 to 0.81)	0.73 (0.68 to 0.77)	0.76 (0.74 to 0.78)	0.78 (0.75 to 0.81)	0.78 (0.75 to 0.81)	0.76 (0.74 to 0.77)
Asian	0.63 (0.56 to 0.71)	0.85 (0.73 to 1.00)	0.78 (0.66 to 0.92)	0.78 (0.73 to 0.83)	0.82 (0.75 to 0.88)	0.80 (0.75 to 0.85)	0.72 (0.69 to 0.75)
***P* for Interaction**	1.48×10^{-6}	0.05	0.003	9.97×10^{-9}	1.60×10^{-7}	0.02	6.39×10^{-7}

Adjusted for age, sex (if applicable), biological (histology type, grade, TNM stage, LVI, comorbidity; ER, PR, HER2 for breast cancer; CA 125 for ovarian cancer; PSA and Gleason grade for prostate cancer; CA 19-9 for pancreatic cancer; AFP, Fibrosis Score, INR for liver cancer; separate tumor nodules for lung cancer; primary site, CEA, circumferential resection margin for colorectal cancer), treatment factors (surgery, chemotherapy, interval between diagnosis to first treatment; endocrine therapy, radiation, immunotherapy, if applicable) and access to care (insurance, facility type, region, urban/rural residence, distance to care, year of diagnosis). *p* values for interaction between racial/ethnic groups and SES groups were derived from log likelihood test. Abbreviation: HR, Hazard Ratio; CI, Confidence Interval.

Supplementary Table S12. Multivariable-adjusted HRs and 95% CIs for 3-year Mortality Associated with Race/ethnicity According to Insurance Status in Major Cancer Types.

	HR (95% CI)						
	**Breast Cancer**	**Ovarian Cancer**	**Prostate Cancer**	**Colorectal Cancer**	**Pancreatic Cancer**	**Liver Cancer**	**Lung Cancer**
**Private Insurance**							
NH White	1.00 (reference)	1.00 (reference)	1.00 (reference)	1.00 (reference)	1.00 (reference)	1.00 (reference)	1.00 (reference)
NH Black	1.29 (1.26 to 1.33)	1.23 (1.16 to 1.30)	1.18 (1.13 to 1.24)	1.08 (1.05 to 1.10)	1.00 (0.97 to 1.03)	0.98 (0.94 to 1.02)	0.96 (0.94 to 0.97)
Hispanic	0.87 (0.83 to 0.92)	0.83 (0.77 to 0.90)	0.89 (0.82 to 0.97)	0.88 (0.84 to 0.91)	0.87 (0.84 to 0.91)	0.81 (0.77 to 0.84)	0.82 (0.80 to 0.84)
Asian	0.82 (0.76 to 0.87)	0.88 (0.80 to 0.97)	0.78 (0.69 to 0.89)	0.87 (0.83 to 0.91)	0.88 (0.84 to 0.93)	0.85 (0.81 to 0.90)	0.70 (0.68 to 0.72)
**Not Insured**							
NH White	1.00 (reference)	1.00 (reference)	1.00 (reference)	1.00 (reference)	1.00 (reference)	1.00 (reference)	1.00 (reference)
NH Black	0.98 (0.92 to 1.06)	1.04 (0.91 to 1.19)	0.84 (0.75 to 0.94)	0.97 (0.92 to 1.02)	0.90 (0.84 to 0.96)	0.96 (0.90 to 1.03)	0.85 (0.83 to 0.88)
Hispanic	0.59 (0.54 to 0.65)	0.59 (0.51 to 0.67)	0.64 (0.56 to 0.74)	0.66 (0.62 to 0.71)	0.66 (0.61 to 0.72)	0.78 (0.72 to 0.84)	0.61 (0.58 to 0.64)
Asian	0.65 (0.55 to 0.78)	0.58 (0.45 to 0.74)	0.54 (0.39 to 0.75)	0.75 (0.67 to 0.84)	0.68 (0.58 to 0.78)	0.79 (0.71 to 0.88)	0.55 (0.51 to 0.60)
**Medicaid**							
NH White	1.00 (reference)	1.00 (reference)	1.00 (reference)	1.00 (reference)	1.00 (reference)	1.00 (reference)	1.00 (reference)
NH Black	1.13 (1.08 to 1.18)	0.97 (0.88 to 1.07)	0.99 (0.90 to 1.08)	1.01 (0.97 to 1.05)	0.93 (0.88 to 0.98)	0.93 (0.89 to 0.98)	0.89 (0.87 to 0.91)
Hispanic	0.68 (0.64 to 0.72)	0.69 (0.62 to 0.77)	0.65 (0.57 to 0.74)	0.73 (0.69 to 0.77)	0.78 (0.73 to 0.83)	0.80 (0.76 to 0.85)	0.70 (0.68 to 0.73)
Asian	0.63 (0.56 to 0.70)	0.79 (0.66 to 0.94)	0.62 (0.50 to 0.77)	0.70 (0.64 to 0.76)	0.70 (0.63 to 0.77)	0.77 (0.71 to 0.82)	0.62 (0.59 to 0.66)
**Medicare**							
NH White	1.00 (reference)	1.00 (reference)	1.00 (reference)	1.00 (reference)	1.00 (reference)	1.00 (reference)	1.00 (reference)
NH Black	1.12 (1.09 to 1.14)	1.13 (1.09 to 1.18)	1.27 (1.24 to 1.31)	1.05 (1.03 to 1.06)	1.00 (0.98 to 1.02)	0.98 (0.94 to 1.01)	0.95 (0.94 to 0.96)
Hispanic	0.77 (0.73 to 0.80)	0.90 (0.84 to 0.96)	0.81 (0.77 to 0.85)	0.83 (0.81 to 0.85)	0.88 (0.85 to 0.90)	0.83 (0.80 to 0.86)	0.84 (0.83 to 0.86)
Asian	0.70 (0.65 to 0.75)	0.84 (0.75 to 0.93)	0.73 (0.67 to 0.80)	0.80 (0.77 to 0.84)	0.96 (0.92 to 1.00)	0.78 (0.74 to 0.82)	0.75 (0.73 to 0.77)
***P* for Interaction**	$< 2.20\times10^{-16}$	4.47×10^{-11}	$< 2.20\times10^{-16}$	$< 2.20\times10^{-16}$	$< 2.20\times10^{-16}$	9.66×10^{-4}	$< 2.20\times10^{-16}$

Adjusted for age, sex (if applicable), biological (histology type, grade, TNM stage, LVI, comorbidity; ER, PR, HER2 for breast cancer; CA 125 for ovarian cancer; PSA and Gleason grade for prostate cancer; CA 19-9 for pancreatic cancer; AFP, Fibrosis Score, INR for liver cancer; separate tumor nodules for lung cancer;

primary site, CEA, circumferential resection margin for colorectal cancer), treatment factors (surgery, chemotherapy, interval between diagnosis to first treatment; endocrine therapy, radiation, immunotherapy, if applicable) and access to care (education, income, facility type, region, urban/rural residence, distance to care, year of diagnosis). *p* values for interaction between racial/ethnic groups and insurance groups were derived from log likelihood test. Cancer patients with unspecified government insurance or unknown insurance status were not included in this analysis. Abbreviation: HR, Hazard Ratio; CI, Confidence Interval.

Supplementary Table S13. Multivariable-adjusted Hazard Ratio (HR) and 95% CIs for 3-year Mortality Associated with Race/ethnicity According to Treating Facility Type in Major Cancer Types.

	HR (95% CI)						
	**Breast Cancer**	**Ovarian Cancer**	**Prostate Cancer**	**Colorectal Cancer**	**Pancreatic Cancer**	**Liver Cancer**	**Lung Cancer**
**Academic/Research Program**							
NH White	1.00 (reference)	1.00 (reference)	1.00 (reference)	1.00 (reference)	1.00 (reference)	1.00 (reference)	1.00 (reference)
NH Black	1.16 (1.13 to 1.19)	1.16 (1.11 to 1.22)	1.21 (1.17 to 1.26)	1.02 (0.99 to 1.04)	1.00 (0.98 to 1.02)	0.98 (0.95 to 1.01)	0.95 (0.94 to 0.96)
Hispanic	0.76 (0.72 to 0.80)	0.79 (0.74 to 0.84)	0.77 (0.72 to 0.83)	0.80 (0.77 to 0.82)	0.82 (0.79 to 0.85)	0.79 (0.76 to 0.82)	0.77 (0.76 to 0.79)
Asian	0.73 (0.68 to 0.79)	0.82 (0.75 to 0.91)	0.71 (0.64 to 0.79)	0.81 (0.78 to 0.85)	0.90 (0.86 to 0.94)	0.77 (0.74 to 0.81)	0.70 (0.68 to 0.72)
**Community**							
NH White	1.00 (reference)	1.00 (reference)	1.00 (reference)	1.00 (reference)	1.00 (reference)	1.00 (reference)	1.00 (reference)
NH Black	1.16 (1.10 to 1.22)	1.12 (0.99 to 1.25)	1.09 (1.03 to 1.17)	1.04 (1.00 to 1.08)	1.01 (0.96 to 1.07)	0.93 (0.85 to 1.00)	0.95 (0.93 to 0.97)
Hispanic	0.73 (0.66 to 0.80)	0.82 (0.70 to 0.96)	0.68 (0.60 to 0.77)	0.80 (0.76 to 0.86)	0.82 (0.76 to 0.89)	0.81 (0.74 to 0.89)	0.85 (0.81 to 0.89)
Asian	0.73 (0.64 to 0.82)	0.75 (0.60 to 0.94)	0.67 (0.55 to 0.80)	0.75 (0.69 to 0.81)	0.88 (0.80 to 0.98)	0.80 (0.70 to 0.90)	0.74 (0.70 to 0.78)
**Comprehensive Community**							
NH White	1.00 (reference)	1.00 (reference)	1.00 (reference)	1.00 (reference)	1.00 (reference)	1.00 (reference)	1.00 (reference)
NH Black	1.16 (1.13 to 1.19)	1.12 (1.06 to 1.18)	1.23 (1.19 to 1.27)	1.05 (1.03 to 1.07)	0.98 (0.96 to 1.01)	0.95 (0.91 to 0.99)	0.94 (0.93 to 0.95)
Hispanic	0.78 (0.74 to 0.81)	0.83 (0.78 to 0.89)	0.81 (0.76 to 0.86)	0.83 (0.81 to 0.86)	0.88 (0.85 to 0.91)	0.82 (0.79 to 0.86)	0.82 (0.80 to 0.84)
Asian	0.68 (0.63 to 0.73)	0.85 (0.76 to 0.94)	0.73 (0.66 to 0.81)	0.79 (0.75 to 0.82)	0.85 (0.81 to 0.90)	0.79 (0.75 to 0.84)	0.72 (0.70 to 0.74)
**Integrated Network**							
NH White	1.00 (reference)	1.00 (reference)	1.00 (reference)	1.00 (reference)	1.00 (reference)	1.00 (reference)	1.00 (reference)
NH Black	1.22 (1.16 to 1.28)	1.13 (1.04 to 1.24)	1.20 (1.13 to 1.29)	1.04 (1.00 to 1.07)	0.97 (0.93 to 1.01)	0.97 (0.91 to 1.02)	0.93 (0.91 to 0.95)
Hispanic	0.62 (0.57 to 0.68)	0.74 (0.65 to 0.84)	0.80 (0.71 to 0.89)	0.74 (0.70 to 0.78)	0.78 (0.74 to 0.83)	0.84 (0.78 to 0.90)	0.75 (0.72 to 0.78)
Asian	0.83 (0.71 to 0.97)	0.75 (0.59 to 0.94)	0.80 (0.64 to 1.01)	0.79 (0.72 to 0.87)	0.93 (0.83 to 1.04)	0.87 (0.78 to 0.96)	0.70 (0.66 to 0.74)
***P* for Interaction**	7.53×10^{-8}	0.29	5.84×10^{-6}	5.91×10^{-3}	1.50×10^{-4}	0.10	2.12×10^{-8}

Adjusted for age, sex (if applicable), biological (histology type, grade, TNM stage, LVI, comorbidity; ER, PR, HER2 for breast cancer; CA 125 for ovarian cancer; PSA and Gleason grade for prostate cancer; CA 19-9 for pancreatic cancer; AFP, Fibrosis Score, INR for liver cancer; separate tumor nodules for lung cancer; primary site, CEA, circumferential resection margin for colorectal cancer), treatment factors (surgery, chemotherapy, interval between diagnosis to first treatment; endocrine therapy, radiation, immunotherapy, if applicable) and access to care (education, income, insurance, region, urban/rural residence, distance to care, year

of diagnosis). *p* values for interaction between racial/ethnic groups and facility types were derived from log likelihood test. Cancer patients with unknown treating facility were not included in this analysis. Abbreviation: HR, Hazard Ratio; CI, Confidence Interval.

•

Supplementary Table S14. Multivariable-adjusted HRs and 95% CIs for 3-year Mortality Associated with Race/ethnicity According to Region in Major Cancer Types.

	HR (95% CI)						
	**Breast Cancer**	**Ovarian Cancer**	**Prostate Cancer**	**Colorectal Cancer**	**Pancreatic Cancer**	**Liver Cancer**	**Lung Cancer**
**Northeast**							
NH White	1.00 (reference)	1.00 (reference)	1.00 (reference)	1.00 (reference)	1.00 (reference)	1.00 (reference)	1.00 (reference)
NH Black	1.13 (1.08 to 1.17)	1.12 (1.03 to 1.21)	1.12 (1.07 to 1.19)	1.02 (0.99 to 1.06)	0.98 (0.94 to 1.02)	0.93 (0.89 to 0.98)	0.92 (0.90 to 0.94)
Hispanic	0.74 (0.69 to 0.80)	0.76 (0.68 to 0.84)	0.73 (0.66 to 0.80)	0.78 (0.74 to 0.82)	0.78 (0.74 to 0.82)	0.72 (0.68 to 0.76)	0.74 (0.72 to 0.77)
Asian	0.69 (0.62 to 0.77)	0.66 (0.56 to 0.77)	0.73 (0.62 to 0.86)	0.73 (0.69 to 0.78)	0.78 (0.72 to 0.84)	0.71 (0.66 to 0.76)	0.62 (0.59 to 0.64)
**Midwest**							
NH White	1.00 (reference)	1.00 (reference)	1.00 (reference)	1.00 (reference)	1.00 (reference)	1.00 (reference)	1.00 (reference)
NH Black	1.09 (1.05 to 1.13)	1.09 (1.01 to 1.17)	1.17 (1.12 to 1.23)	1.00 (0.97 to 1.03)	0.96 (0.93 to 1.00)	0.92 (0.87 to 0.96)	0.91 (0.90 to 0.93)
Hispanic	0.76 (0.68 to 0.84)	0.98 (0.86 to 1.13)	0.70 (0.62 to 0.79)	0.80 (0.75 to 0.86)	0.87 (0.81 to 0.93)	0.92 (0.86 to 0.99)	0.85 (0.81 to 0.89)
Asian	0.72 (0.63 to 0.83)	0.89 (0.73 to 1.09)	0.76 (0.63 to 0.92)	0.74 (0.68 to 0.80)	0.90 (0.82 to 0.99)	0.82 (0.76 to 0.90)	0.71 (0.68 to 0.75)
**South**							
NH White	1.00 (reference)	1.00 (reference)	1.00 (reference)	1.00 (reference)	1.00 (reference)	1.00 (reference)	1.00 (reference)
NH Black	1.20 (1.18 to 1.23)	1.17 (1.12 to 1.22)	1.24 (1.20 to 1.28)	1.05 (1.03 to 1.06)	1.00 (0.98 to 1.02)	1.01 (0.98 to 1.04)	0.96 (0.95 to 0.97)
Hispanic	0.70 (0.67 to 0.74)	0.76 (0.71 to 0.81)	0.75 (0.71 to 0.80)	0.76 (0.74 to 0.78)	0.80 (0.77 to 0.83)	0.79 (0.77 to 0.82)	0.77 (0.75 to 0.78)
Asian	0.74 (0.66 to 0.82)	0.81 (0.70 to 0.94)	0.72 (0.61 to 0.85)	0.80 (0.75 to 0.86)	0.90 (0.83 to 0.97)	0.81 (0.76 to 0.86)	0.73 (0.70 to 0.76)
**West**							
NH White	1.00 (reference)	1.00 (reference)	1.00 (reference)	1.00 (reference)	1.00 (reference)	1.00 (reference)	1.00 (reference)
NH Black	1.22 (1.14 to 1.30)	1.24 (1.11 to 1.40)	1.33 (1.22 to 1.44)	1.08 (1.03 to 1.13)	1.04 (0.98 to 1.10)	0.91 (0.85 to 0.97)	0.99 (0.96 to 1.02)
Hispanic	0.83 (0.79 to 0.88)	0.87 (0.81 to 0.94)	0.87 (0.81 to 0.93)	0.91 (0.88 to 0.95)	0.93 (0.90 to 0.97)	0.89 (0.85 to 0.92)	0.89 (0.86 to 0.91)
Asian	0.73 (0.69 to 0.78)	0.90 (0.83 to 0.99)	0.72 (0.66 to 0.79)	0.84 (0.81 to 0.87)	0.93 (0.90 to 0.97)	0.82 (0.79 to 0.86)	0.76 (0.74 to 0.78)
***P** for Interaction*	1.86×10^{-12}	4.39×10^{-8}	9.58×10^{-14}	$< 2.20\times10^{-16}$	$< 2.20\times10^{-16}$	$< 2.20\times10^{-16}$	$< 2.20\times10^{-16}$

Adjusted for age, sex (if applicable), biological (histology type, grade, TNM stage, LVI, comorbidity; ER, PR, HER2 for breast cancer; CA 125 for ovarian cancer; PSA and Gleason grade for prostate cancer; CA 19-9 for pancreatic cancer; AFP, Fibrosis Score, INR for liver cancer; separate tumor nodules for lung cancer;

primary site, CEA, circumferential resection margin for colorectal cancer), treatment factors (surgery, chemotherapy, interval between diagnosis to first treatment; endocrine therapy, radiation, immunotherapy, if applicable) and access to care (education, income, insurance, facility type, urban/rural residence, distance to care, year of diagnosis). *P* values for interaction between racial/ethnic groups and regions were derived from log likelihood test. Cancer patients with unknown region information were not included in this analysis. Abbreviation: HR, Hazard Ratio; CI, Confidence Interval.

•

Supplementary Table S15. Multivariable-adjusted HRs and 95% CIs for 3-year Mortality Associated with Race/ethnicity According to Urban/rural Residence in Major Cancer Types.

	HR (95% CI)						
	**Breast Cancer**	**Ovarian Cancer**	**Prostate Cancer**	**Colorectal Cancer**	**Pancreatic Cancer**	**Liver Cancer**	**Lung Cancer**
	**Metro Residence**						
NH White	1.00 (reference)	1.00 (reference)	1.00 (reference)	1.00 (reference)	1.00 (reference)	1.00 (reference)	1.00 (reference)
NH Black	1.16 (1.14 to 1.18)	1.12 (1.09 to 1.16)	1.20 (1.17 to 1.23)	1.04 (1.02 to 1.05)	0.98 (0.97 to 1.00)	0.96 (0.94 to 0.98)	0.94 (0.93 to 0.95)
Hispanic	0.75 (0.73 to 0.77)	0.80 (0.76 to 0.83)	0.77 (0.74 to 0.81)	0.80 (0.79 to 0.82)	0.83 (0.81 to 0.85)	0.80 (0.78 to 0.82)	0.78 (0.77 to 0.79)
Asian	0.71 (0.68 to 0.75)	0.79 (0.74 to 0.85)	0.69 (0.64 to 0.74)	0.79 (0.76 to 0.81)	0.88 (0.85 to 0.91)	0.78 (0.76 to 0.81)	0.71 (0.69 to 0.72)
	**Urban Residence**						
NH White	1.00 (reference)	1.00 (reference)	1.00 (reference)	1.00 (reference)	1.00 (reference)	1.00 (reference)	1.00 (reference)
NH Black	1.25 (1.19 to 1.32)	1.31 (1.19 to 1.44)	1.22 (1.14 to 1.30)	1.07 (1.03 to 1.12)	1.04 (0.99 to 1.09)	0.96 (0.89 to 1.04)	0.94 (0.92 to 0.97)
Hispanic	0.88 (0.78 to 0.99)	0.86 (0.73 to 1.02)	0.80 (0.68 to 0.93)	0.87 (0.80 to 0.94)	0.83 (0.77 to 0.91)	0.89 (0.82 to 0.97)	0.92 (0.87 to 0.97)
Asian	0.73 (0.55 to 0.96)	0.97 (0.69 to 1.37)	0.65 (0.41 to 1.04)	0.89 (0.75 to 1.06)	0.85 (0.71 to 1.02)	1.03 (0.87 to 1.23)	0.79 (0.71 to 0.87)
	**Rural Residence**						
NH White	1.00 (reference)	1.00 (reference)	1.00 (reference)	1.00 (reference)	1.00 (reference)	1.00 (reference)	1.00 (reference)
NH Black	1.25 (1.07 to 1.45)	1.13 (0.86 to 1.48)	1.27 (1.05 to 1.54)	1.03 (0.92 to 1.16)	1.06 (0.94 to 1.21)	0.77 (0.60 to 1.00)	0.96 (0.90 to 1.02)
Hispanic	0.89 (0.56 to 1.42)	0.34 (0.15 to 0.78)	0.60 (0.28 to 1.27)	0.97 (0.75 to 1.27)	0.92 (0.69 to 1.23)	0.89 (0.63 to 1.25)	0.83 (0.67 to 1.03)
Asian	0.29 (0.04 to 2.09)	0.88 (0.21 to 3.64)	3.34 (0.83 to 13.45)	1.10 (0.57 to 2.12)	0.81 (0.38 to 1.71)	1.27 (0.59 to 2.75)	1.15 (0.79 to 1.69)
***P* for Interaction**	2.81×10^{-5}	7.77×10^{-3}	0.15	1.08×10^{-3}	0.01	7.41×10^{-3}	4.33×10^{-8}

Adjusted for age, sex (if applicable), biological (histology type, grade, TNM stage, LVI, comorbidity; ER, PR, HER2 for breast cancer; CA 125 for ovarian cancer; PSA and Gleason grade for prostate cancer; CA 19-9 for pancreatic cancer; AFP, Fibrosis Score, INR for liver cancer; separate tumor nodules for lung cancer; primary site, CEA, circumferential resection margin for colorectal cancer), treatment factors (surgery, chemotherapy, interval between diagnosis to first treatment; endocrine therapy, radiation, immunotherapy, if applicable) and access to care (education, income, insurance, facility type, region, distance to care, year of diagnosis).*P* values for interaction between racial/ethnic groups and urban/rural residence were derived from log likelihood test. Cancer patients with unknown urban/rural residence information were not included in this analysis.Abbreviation: HR, Hazard Ratio; CI, Confidence Interval.

Supplementary Table S16. HRs and 95% CIs for Total Mortality Associated with Race/ethnicity (sensitivity analysis excluding individuals with missing information on access-to-care related factors).

Race/ethnicity	HR (95% CI)						
	Breast Cancer	Ovarian Cancer	Prostate Cancer	Colorectal Cancer	Pancreatic Cancer	Liver Cancer	Lung Cancer
NH-white	1.00 (reference)	1.00 (reference)	1.00 (reference)	1.00 (reference)	1.00 (reference)	1.00 (reference)	1.00 (reference)
NH-black	1.26 (1.24 to 1.27)	1.20 (1.17 to 1.23)	1.31 (1.29 to 1.33)	1.11 (1.10 to 1.12)	1.03 (1.01 to 1.04)	1.00 (0.98 to 1.02)	0.97 (0.97 to 0.98)
Hispanic	0.82 (0.80 to 0.84)	0.84 (0.81 to 0.87)	0.84 (0.82 to 0.86)	0.86 (0.84 to 0.87)	0.86 (0.84 to 0.88)	0.83 (0.81 to 0.84)	0.80 (0.79 to 0.81)
Asian	0.68 (0.66 to 0.70)	0.82 (0.77 to 0.86)	0.68 (0.65 to 0.71)	0.79 (0.77 to 0.81)	0.87 (0.84 to 0.90)	0.76 (0.74 to 0.78)	0.72 (0.70 to 0.73)

Abbreviation: HR, Hazard Ratio; CI, Confidence Interval.* Adjusted for age, sex (if applicable), biological (histology type, grade, TNM stage, LVI, comorbidity; ER, PR, HER2 for breast cancer; CA 125 for ovarian cancer; PSA and Gleason grade for prostate cancer; CA 19-9 for pancreatic cancer; AFP, Fibrosis Score, INR for liver cancer; separate tumor nodules for lung cancer; primary site, CEA, circumferential resection margin for colorectal cancer), treatment factors (surgery, chemotherapy, interval between diagnosis to first treatment; endocrine therapy, radiation, immunotherapy, if applicable) and year of diagnosis.

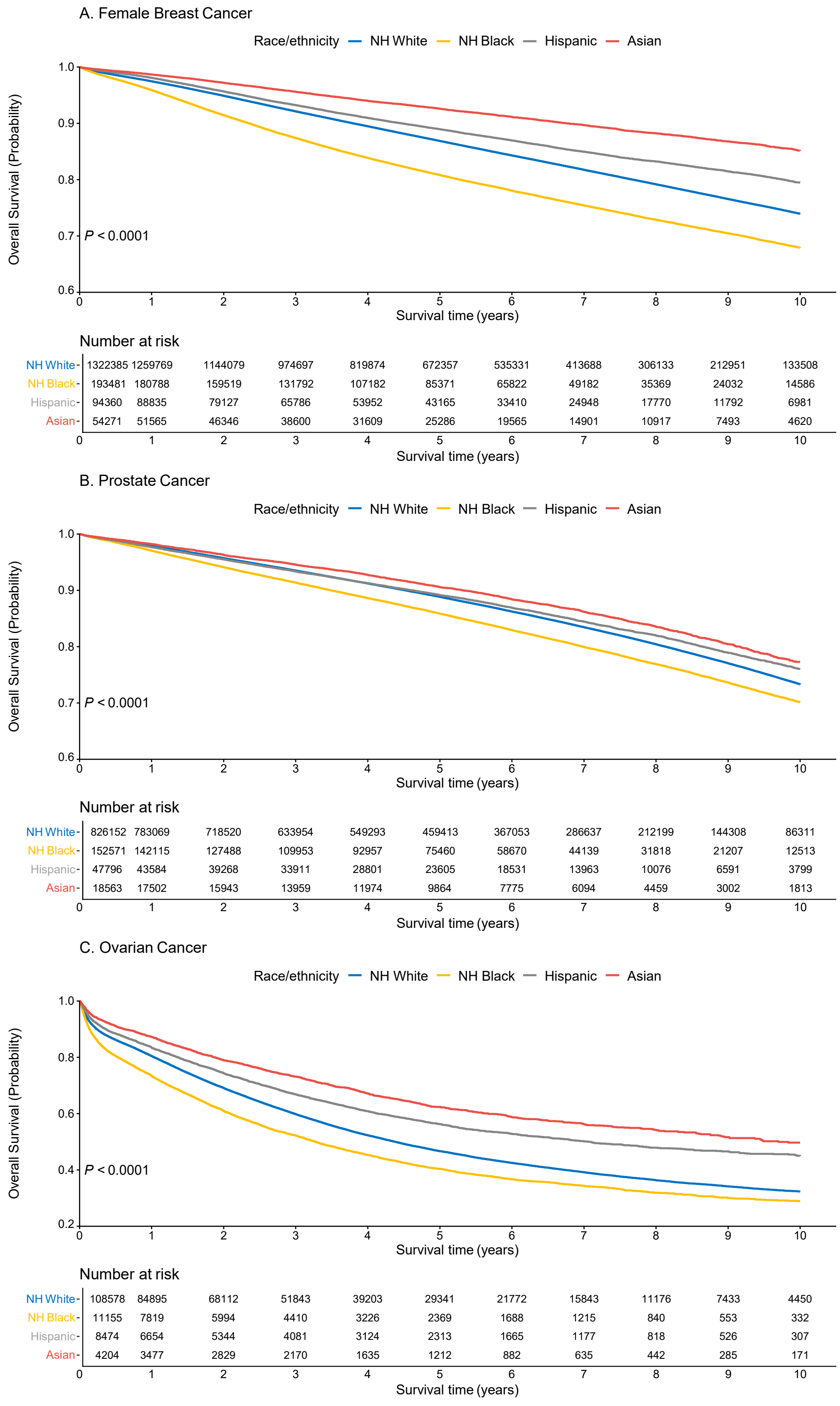

Supplementary Figure S1. Crude Overall Survival of Sex-specific Cancers

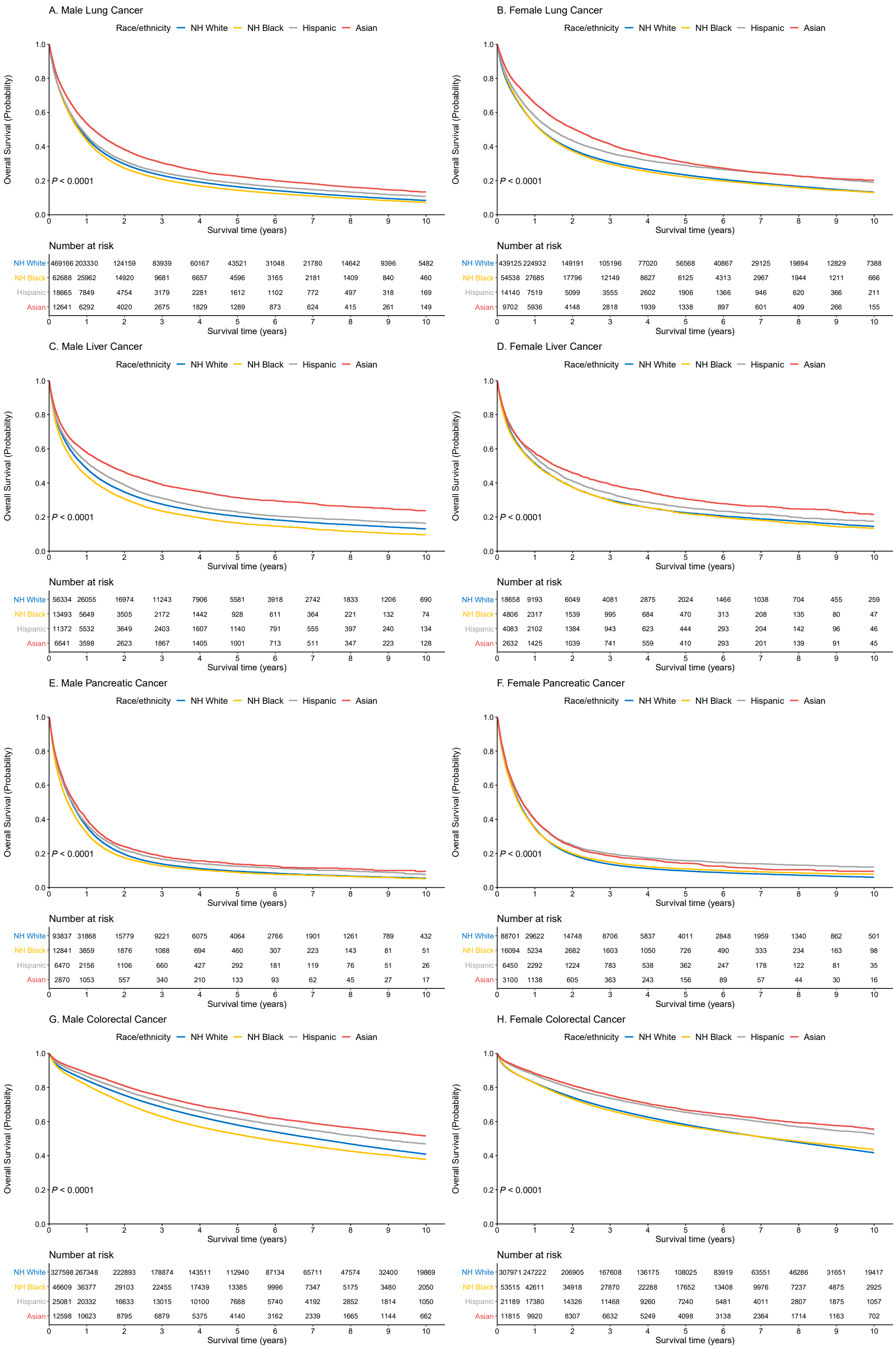

Supplementary Figure S2. Crude Overall Survival of Lung, Liver, Pancreatic and Colorectal Cancers by Sex

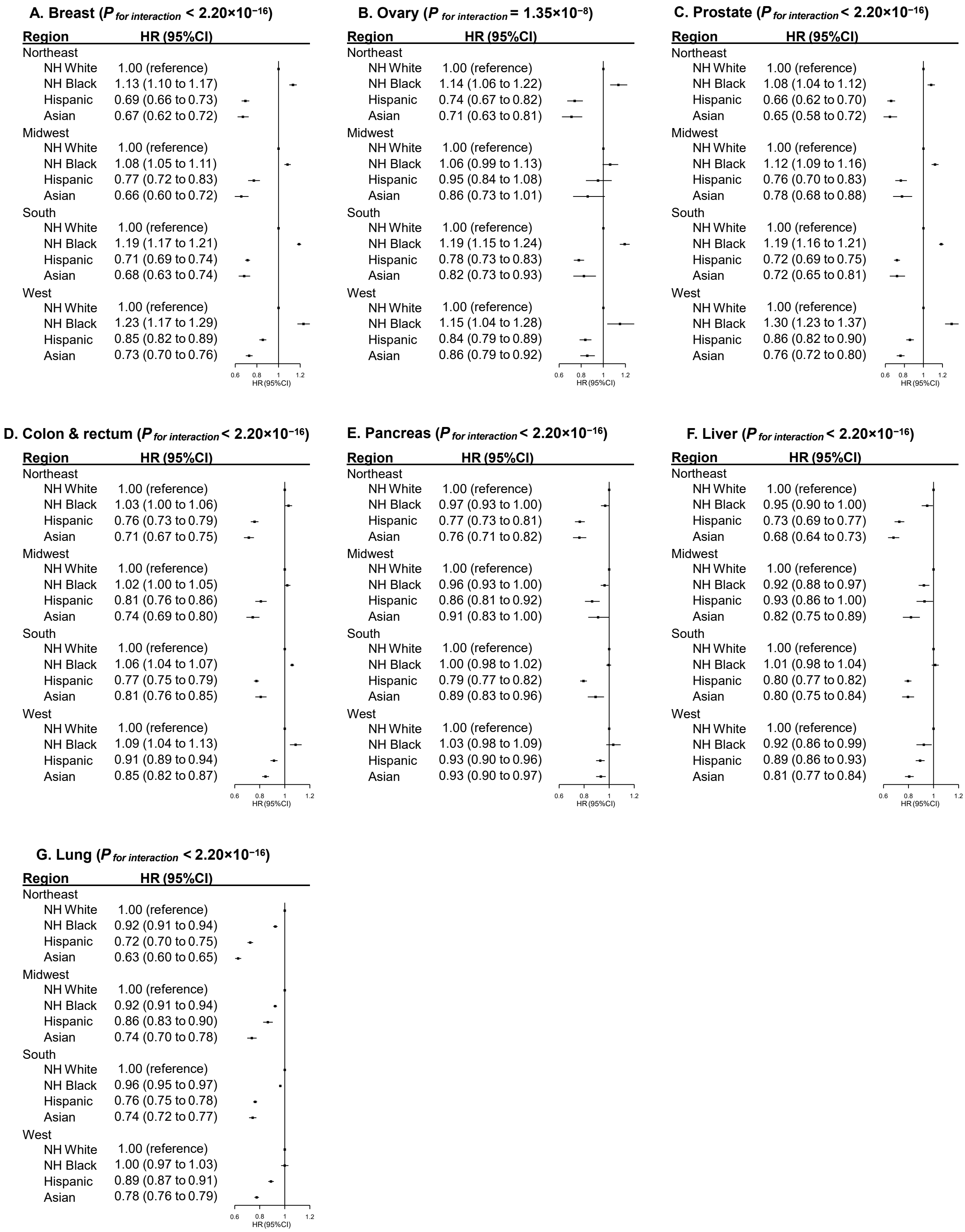

Supplementary Figure S3. HRs and 95% CIs for Total Mortality Associated with Race/ethnicity According to Region in Major Cancer Types.

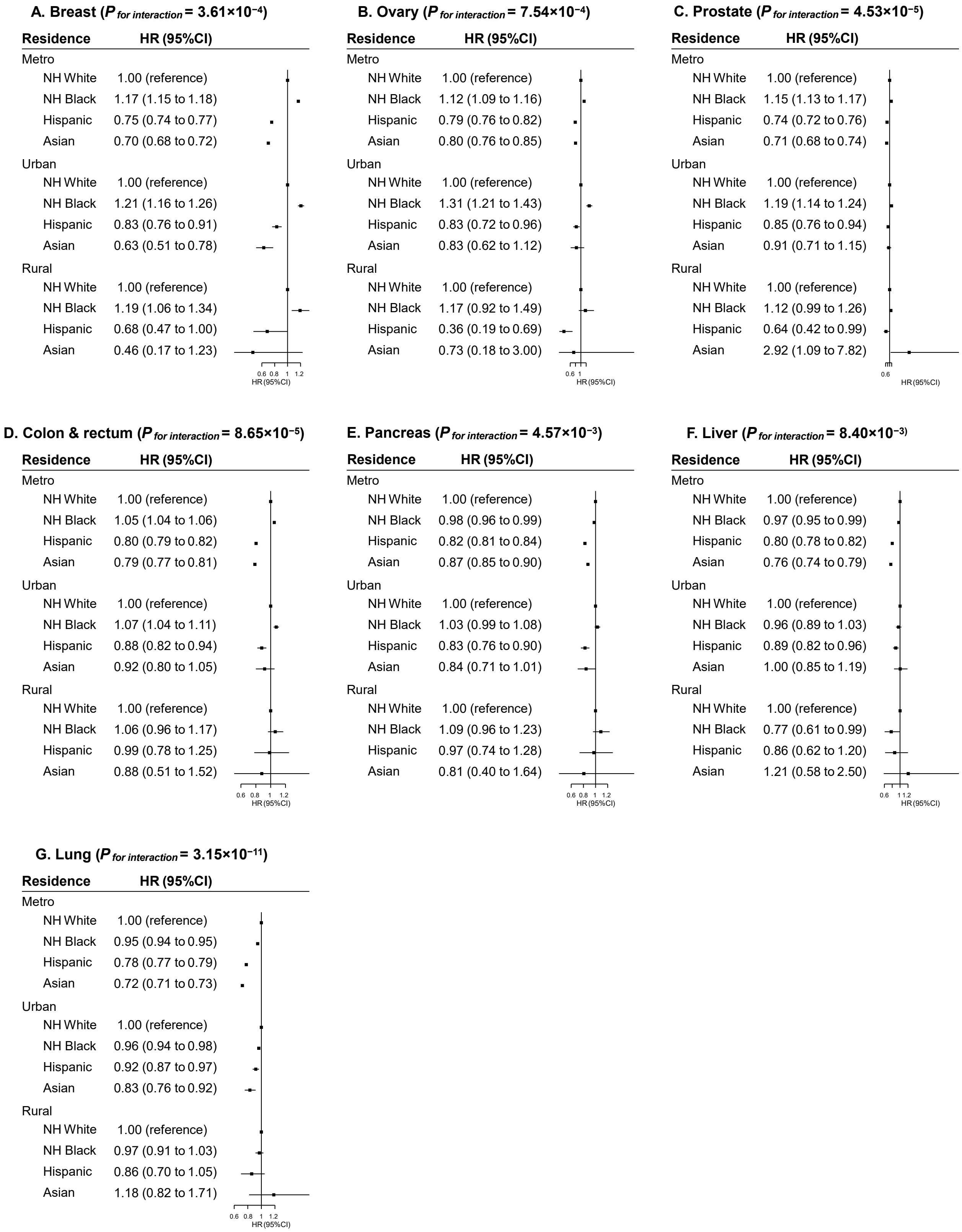

Supplementary Figure S4. HRs and 95% CIs for Total Mortality Associated with Race/ethnicity According to Urban/rural Residence in Major Cancer Types.

Supplementary Figure Legends

Supplementary Figure S1. Crude Overall Survival of Sex-specific Cancers. (A) Female breast cancer (N=1,664,497); (B) Ovarian cancer (N=132,411); (C) Prostate cancer (N=1,045,082). *P* value is from the log-rank test.

Supplementary Figure S2. Crude Overall Survival of Lung, Liver, Pancreatic and Colorectal Cancers by Sex. (A) Male lung cancer (N=563,160); (B) Female lung cancer (N=517,505); (C) Male liver cancer (N=87,840); (D) Female liver cancer (N=30,179); (E) Male pancreatic cancer (N=116,018); (F) Female pancreatic cancer (N=114,345); (G) Male colorectal cancer (N=411,886); (H) Female colorectal cancer (N=394,490). *P* value is from the log-rank test.

Supplementary Figure S3. The multivariable-adjusted HRs and 95% CIs for total mortality associated with race/ethnicity according to region in major cancer types. The HRs and 95% CIs were adjusted for age, sex (if applicable), biology (histology type, grade, TNM stage, LVI, comorbidity; ER, PR, HER2 for breast cancer; CA 125 for ovarian cancer; PSA and Gleason grade for prostate cancer; CA 19-9 for pancreatic cancer; AFP, Fibrosis Score, INR for liver cancer; separate tumor nodules for lung cancer; primary site, CEA, circumferential resection margin for colorectal cancer), treatment factors (surgery, chemotherapy, interval between diagnosis to first treatment; and endocrine therapy, radiation, immunotherapy, if applicable) and access to care (education, income, insurance, treating facility types, urban/rural residence, distance to care, and year of diagnosis). *P* values for interaction between racial/ethnic groups and regions were derived from log likelihood tests. Cancer patients with unknown region information were not included in this analysis. Abbreviation: HR, Hazard Ratio; CI, Confidence Interval.

Supplementary Figure S4. The multivariable-adjusted HRs and 95% CIs for total mortality associated with race/ethnicity according to urban/rural residence in major cancer types. The HRs and 95% CIs were adjusted for age, sex (if applicable), biology (histology type, grade, TNM stage, LVI, comorbidity; ER, PR, HER2 for breast cancer; CA 125 for ovarian cancer; PSA and Gleason grade for prostate cancer; CA 19-9 for pancreatic cancer; AFP, Fibrosis Score, INR for liver cancer; separate tumor nodules for lung cancer; primary site, CEA, circumferential resection margin for colorectal cancer), treatment factors (surgery, chemotherapy, interval between diagnosis to first treatment; and endocrine therapy, radiation, immunotherapy, if applicable) and access to care (education, income, insurance, treating facility type, region, distance to care, and year of diagnosis). *P* values for interaction between racial/ethnic groups and residence were derived from log likelihood tests. Cancer patients with unknown residence information were not included in this analysis. Abbreviation: HR, Hazard Ratio; CI, Confidence Interval.
